# Culturally grounded health communication to support self-care among pregnant women with diabetes: Development and content validation of an educational *cordel* in Brazil

**DOI:** 10.1371/journal.pone.0357959

**Published:** 2026-09-11

**Authors:** Francisca Édla Santos Leite Gurgel, Raimunda Magalhães da Silva, Jonas Loiola Gonçalves, Cidianna Emanuelly Melo do Nascimento, Rita Neuma Dantas Cavalcante de Abreu, Maria Helena de Carvalho Valente Presado, Suely Kazue Nagahashi Marie

**Affiliations:** 1 University of Fortaleza. Avenida Washington Soares, Edson Queiroz, Fortaleza, Ceará, Brazil; 2 School of Medicine, University of São Paulo, São Paulo, São Paulo, Brazil; 3 State University of Ceará, Fortaleza, Ceará, Brazil; 4 Lisbon School of Nursing, Lisbon, Portugal; Federal University of Santa Maria: Universidade Federal de Santa Maria, BRAZIL

## Abstract

**Background:**

Diabetes in pregnancy requires sustained self-care and clear guidance across hospital, home, and primary care settings. Conventional educational materials may be less accessible to women facing social vulnerability or limited health literacy. Culturally grounded communication may improve the understandability and relevance of discharge guidance.

**Methods:**

We conducted a methodological study in two public tertiary maternity hospitals in Fortaleza, Brazil, to develop and content-validate an educational *cordel*, a traditional Brazilian form of rhymed popular literature. The material was developed through a structured evidence search, collaboration with health researchers and *cordelistas*, and pilot testing. It was evaluated by 22 hospitalized pregnant women with gestational diabetes mellitus or pre-existing type 2 diabetes and by 40 expert judges. Item-level Content Validity Indices (I-CVIs), scale-level CVI by averaging item values (S-CVI/Ave), Cronbach’s alpha, and Kendall’s coefficient of concordance were calculated.

**Results:**

All target-population items achieved an I-CVI of 1.00, and the S-CVI/Ave was 1.00. The evaluation responses showed modest internal consistency (raw alpha = 0.67; standardized alpha = 0.66). Among expert judges, I-CVIs ranged from 0.95 to 1.00 and the S-CVI/Ave was 0.99. Internal consistency of expert responses was high (raw alpha = 0.96; standardized alpha = 0.97), whereas Kendall’s W was 0.039, indicating weak concordance in the relative ranking of items despite high absolute favorable agreement.

**Conclusion:**

The educational *cordel* showed strong preliminary evidence of content validity, cultural relevance, and acceptability for hospitalized pregnant women with diabetes. The findings support further testing in larger and more diverse samples, using standardized measures of understandability and actionability and designs that evaluate effects on knowledge, self-care, continuity of care, and clinical outcomes.

## Introduction

Pregnancy involves substantial physical, psychological, interpersonal, and social changes. Although most pregnancies progress without major complications, pre-existing chronic conditions and adverse social circumstances can increase the risk of maternal and fetal morbidity and mortality [1–6].

High-risk pregnancy may be associated with obesity, diabetes mellitus, hypertension, mental health conditions, substance use, exposure to violence, extremes of maternal age, and barriers to timely care. These clinical and social factors interact and can intensify vulnerability throughout pregnancy and the postpartum period [3,7,8].

Diabetes in pregnancy includes gestational diabetes mellitus (GDM), first recognized during pregnancy, and pre-existing diabetes, including type 2 diabetes mellitus (T2DM). Both conditions require glycemic monitoring and coordinated clinical management to reduce adverse maternal and neonatal outcomes. Their burden is increasing in Brazil and other low- and middle-income countries, where overweight, unhealthy dietary patterns, and structural inequities compound risk [9–13].

A diagnosis that requires hospitalization and continued monitoring may generate fear, anxiety, insecurity, and uncertainty. These experiences can be intensified by low educational attainment, limited access to understandable health information, and separation from family and daily routines [2,14–19].

Self-care in diabetes in pregnancy includes daily decisions regarding diet, physical activity, medication, glycemic monitoring, symptom recognition, and follow-up. The ability to act on these recommendations depends not only on individual motivation but also on health literacy, family support, material resources, and the accessibility of communication provided by health services [20,21].

Conventional education based primarily on technical or prescriptive language may not adequately address these needs. Educational strategies should therefore use clear language, active listening, cultural sensitivity, and recognition of women’s social contexts to strengthen understanding, autonomy, and engagement in care [2,14,17,20].

*Cordel* literature is a traditional Brazilian form of popular communication characterized by rhymed verses, oral performance, and illustrated booklets. Its accessible language, narrative rhythm, and regional symbolism have supported health communication on topics such as HIV prevention, breastfeeding, and dengue [22–24]. These features make *cordel* a potentially useful format for translating technical recommendations into culturally meaningful guidance.

However, evidence remains limited regarding the development and systematic validation of *cordel*-based educational materials for women with diabetes during pregnancy, particularly for use at hospital discharge and during transition to primary care. Establishing content validity and acceptability is a necessary preliminary step before testing effectiveness.

This study aimed to develop and content-validate an educational *cordel* to support self-care and continuity after hospital discharge among hospitalized pregnant women with GDM or pre-existing T2DM, based on evaluation by the target population and expert judges.

## Materials and methods

### Ethical considerations

This study was part of the umbrella project Analysis of Care for High-Risk Pregnant and Postpartum Women. The Research Ethics Committee of the University of Fortaleza and the ethics committees of the two participating institutions approved the study (approval numbers 6,789,730/2024, 6,820,534/2024, and 6,844,667/2024). All participants provided written informed consent before data collection.

### Study design

We conducted a methodological study focused on the development, refinement, and preliminary content validation of a health education technology for pregnant women with diabetes [25].

### Study setting

The study was conducted at Dr. César Cals General Hospital (HGCC) and the Assis Chateaubriand Teaching Maternity Hospital (MEAC), two public tertiary referral hospitals for high-risk pregnancy in Fortaleza, Ceará, northeastern Brazil. Both hospitals provide obstetric emergency, inpatient, and specialist care to women with conditions requiring intensified monitoring, including diabetes.

### Study phases

Development and target-population data collection occurred in 2024; pregnant women were recruited from 4 November to 31 December 2024. Expert-judge validation was completed in 2025. The study comprised five sequential phases.

1. Structured evidence search

A structured evidence search was undertaken to define the educational content. This was a content-development search rather than a systematic review. Peer-reviewed national and international studies and Brazilian technical and policy documents addressing diabetes in pregnancy, health education, self-care, postpartum follow-up, and continuity of care were consulted. Findings were organized into thematic domains and translated into key messages for the *cordel*.

2. Development of the educational *cordel*

Four health researchers worked with experienced *cordelistas* to transform the evidence-based messages into a culturally grounded narrative. Traditional characteristics of *cordel* were retained, including rhymed verses, metric regularity, and thematic organization into stanzas, while clinical content was reviewed for consistency with recommended care for diabetes in pregnancy. The final *cordel* is presented in its original Portuguese in the S1 File to preserve the regional characteristics of its language, rhymes, rhythmic structure, and cultural meaning, while its English version is provided in the S2 File.

Development took 45 days and included script writing, illustration selection, and preparation of the first prototype. Content was discussed and refined in face-to-face and online meetings to translate technical information into clear and engaging language without changing its clinical meaning.

The material addressed diabetes in pregnancy, treatment, healthy eating, physical activity, glycemic monitoring and recording, nursing guidance, postpartum care, possible neonatal complications, and long-term follow-up. Illustrations, colors, and layout were selected to support comprehension and contribute to the visual identity.

3. Pilot testing and refinement

Two pregnant women receiving care at the participating hospitals completed a qualitative pilot test. Guided reading and probing questions explored semantic clarity, language, cultural appropriateness, and comprehension of self-care recommendations. Both women considered the material understandable and relevant. Their suggestions led to minor wording changes and clarification of selected recommendations; no domain, evaluation item, or substantive clinical recommendation was changed. Their evaluations were retained in the final target-population sample, a decision considered in the limitations.

4. Validation with the target population

After refinement, 22 hospitalized pregnant women with GDM or pre-existing T2DM evaluated the *cordel*. They read the complete material and rated items in the domains of objectives, structure and presentation, and relevance. Written suggestions were reviewed by the research team and informed final editorial adjustments.

5. Validation by expert judges

In 2025, 40 expert judges independently evaluated the *cordel* using the same domains. The panel comprised 39 obstetric nurses and one obstetrician. Their ratings were used to estimate item-level and scale-level content validity, response consistency, and concordance.

### Eligibility criteria and sampling

Eligible target-population participants were hospitalized pregnant women aged 18 years or older, diagnosed with GDM or T2DM, admitted for glycemic control, and clinically stable at the time of evaluation. Women with clinical instability or transfer to another tertiary unit during the collection period were excluded.

Eligible expert judges were health professionals with specialist training and at least two years of experience in obstetric or women’s health care. Professionals who were on vacation or personal or medical leave during data collection were excluded.

Convenience sampling was used in both groups because recruitment depended on eligibility and availability within the clinical setting. The sample sizes were determined by the number of eligible participants recruited during the prespecified periods. The analyses were intended to provide preliminary content-validity and response-consistency evidence rather than population-level estimates [25].

### Data collection

Potential participants were screened against the eligibility criteria and received an explanation of the study objectives and procedures. Those who agreed to participate signed two copies of the informed consent form, retaining one copy.

Pregnant women received a kit containing the printed *cordel* and the evaluation form. They were given sufficient time to read the material and complete the form, while a researcher remained available to clarify procedural questions without directing responses. Expert judges received the *cordel* and completed their assessments independently.

The structured evaluation form contained items grouped into three domains: objectives, structure and presentation, and relevance. Responses were recorded on a five-point scale: 1 = totally agree, 2 = partially agree, 3 = neutral, 4 = partially disagree, and 5 = totally disagree. The form was adapted for this study to assess the adequacy, clarity, organization, and perceived usefulness of the educational material. The English-language target-population and expert-judge evaluation forms are provided as S3 File.

### Data analysis

Categorical variables were summarized as counts and percentages. Continuous variables were summarized as mean and standard deviation or median and interquartile range, as appropriate. For each evaluation item, ratings of 1 or 2 were classified as favorable. The item-level Content Validity Index (I-CVI) was calculated as the number of favorable ratings divided by the number of valid responses. The scale-level CVI by the average method (S-CVI/Ave) was calculated as the mean of all I-CVIs. An I-CVI of at least 0.80 was prespecified as acceptable.

Cronbach’s alpha was used to describe internal consistency of responses across evaluation items, and alpha if item deleted was examined. Alpha was interpreted as a property of the evaluation responses, not as a direct measure of the educational material’s effectiveness. For expert judges, Kendall’s coefficient of concordance (W) was also calculated to assess agreement in the relative ordering of item ratings. Missing responses were excluded item by item.

Analyses were performed in R version 4.3.1 using the psych package for reliability analysis, dplyr for data management, and base R functions for statistical calculations. The analytical procedures, including the scoring rules, missing-response handling, and statistical methods, are described above.

## Results

### Pilot test

The two pilot participants considered the *cordel* clear, understandable, culturally appropriate, and relevant. Guided feedback led to minor wording changes and clarification of selected self-care recommendations; no structural changes were required.

### Characteristics of the target population

Twenty-two pregnant women participated: 12 (54.5%) at MEAC and 10 (45.5%) at HGCC. Their mean age was 32.2 years (SD 5.6). Twenty participants (90.9%) self-identified as Black or Brown, 15 (68.2%) were homemakers, and 14 (63.6%) lived in Fortaleza. Median monthly household income was BRL 1,530 (IQR 1,400−2,500), and 15 participants (68.2%) received social benefits (Table 1).

**Table 1:** Sociodemographic and clinical characteristics of pregnant women who evaluated the educational *cordel* (n = 22), Fortaleza, Ceará, Brazil, 2024.

Variable	n	%
**Referring hospital**		
HGCC¹	10	45.5
MEAC²	12	54.5
Age, years, mean ± SD	32.2 ± 5.6	
**Self-reported race/skin color**		
White	2	9.1
Black/Brown	20	90.9
**Occupation**		
Homemaker	15	68.2
Other	7	31.8
**Marital status**		
Single	8	36.4
Married	7	31.8
Others	7	31.8
**Education level**		
Primary	6	27.3
Secondary	14	63.6
Higher	2	9.1
**Place of residence**		
Fortaleza (state capital)	14	63.6
Other municipalities	8	36.4
Monthly household income, BRL, median (IQR)	1,530 (1,400–2,500)	
Receives social benefits (yes)	15	68.2
Number of pregnancies, mean ± SD	2.9 ± 1.4	
Prenatal care initiation, days, mean ± SD	66.8 ± 31.5	
**Site where prenatal care began**		
HGCC	1	4.6
MEAC	2	9.1
Polyclinic	2	9.1
Health center	5	22.7
Primary Health Care Unit	2	9.1
Others	10	45.5
Gestational age, weeks, mean ± SD	30.7 ± 5.8	
Hospital stay, days, median (range)	6.0 (3–29)	
**Diagnosis**		
T2DM³	11	50.0
GDM⁴	11	50.0

^1^HGCC, Dr. César Cals General Hospital; ^2^MEAC, Assis Chateaubriand Teaching Maternity Hospital; ^3^T2DM, type 2 diabetes mellitus; ^4^GDM, gestational diabetes mellitus. Categorical variables are n (%). Continuous variables are mean ± standard deviation or median (interquartile range/range), as indicated.

Eight women (36.4%) were single, seven (31.8%) were married, and seven (31.8%) reported other marital arrangements, including stable unions. Fourteen participants (63.6%) had completed secondary education, six (27.3%) primary education, and two (9.1%) higher education (Table 1).

The mean number of pregnancies was 2.9 (SD 1.4), and prenatal care began at a mean of 66.8 days of gestation (SD 31.5). Participants had initiated prenatal care across maternity hospitals, a polyclinic, primary care services, and other facilities (Table 1).

Mean gestational age at evaluation was 30.7 weeks (SD 5.8), and median hospital stay was 6 days (range 3–29). Eleven participants (50.0%) had GDM and 11 (50.0%) had pre-existing T2DM (Table 1).

#### Target-population evaluation of the educational *cordel.*

In the objectives domain, total agreement ranged from 86.4% to 95.5%. All remaining responses were partial agreement, and no participant selected neutral, partially disagree, or totally disagree. The highest total agreement concerned easy-to-understand language and encouragement of behavioral change (both 95.5%) (Table 2).

**Table 2:** Target-population ratings, item-level content validity, and alpha if item deleted for the educational *cordel* (n = 22), Fortaleza, Ceará, Brazil, 2024.

Item	Response scale, n (%)					I-CVI	Alpha if item deleted
	1	2	3	4	5		
**Objectives**							
The cordel encourages engagement in diabetes mellitus (DM) care	19 (86.4%)	3 (13.6%)	0 (0.0%)	0 (0.0%)	0 (0.0%)	1.00	0.63
It uses easy-to-understand language	21 (95.5%)	1 (4.5%)	0 (0.0%)	0 (0.0%)	0 (0.0%)	1.00	0.66
It contains information and explanations to guide DM care	19 (86.4%)	3 (13.6%)	0 (0.0%)	0 (0.0%)	0 (0.0%)	1.00	0.53
It encourages behavioral changes in daily care related to the disease and pregnancy	21 (95.5%)	1 (4.5%)	0 (0.0%)	0 (0.0%)	0 (0.0%)	1.00	0.70
**Structure and presentation**							
The cordel-style writing is easy to understand	21 (95.5%)	1 (4.5%)	0 (0.0%)	0 (0.0%)	0 (0.0%)	1.00	0.62
The text is clear	21 (95.5%)	1 (4.5%)	0 (0.0%)	0 (0.0%)	0 (0.0%)	1.00	0.64
The text is interesting	22 (100.0%)	0 (0.0%)	0 (0.0%)	0 (0.0%)	0 (0.0%)	1.00	0.62
The suggested content is appropriate	22 (100.0%)	0 (0.0%)	0 (0.0%)	0 (0.0%)	0 (0.0%)	1.00	0.64
The font size is adequate	21 (95.5%)	1 (4.5%)	0 (0.0%)	0 (0.0%)	0 (0.0%)	1.00	0.62
The illustrations help to complement the text	21 (95.5%)	1 (4.5%)	0 (0.0%)	0 (0.0%)	0 (0.0%)	1.00	0.62
**Relevance**							
The material addresses the necessary topics for understanding DM care during pregnancy	21 (95.5%)	1 (4.5%)	0 (0.0%)	0 (0.0%)	0 (0.0%)	1.00	0.68
The material is suitable to assist with DM care at home	21 (95.5%)	1 (4.5%)	0 (0.0%)	0 (0.0%)	0 (0.0%)	1.00	0.64
It allows reflection on mistakes made leading to behavioral change	19 (86.4%)	3 (13.6%)	0 (0.0%)	0 (0.0%)	0 (0.0%)	1.00	0.65
The use of technology is relevant for pregnant women with DM	20 (90.9%)	2 (9.1%)	0 (0.0%)	0 (0.0%)	0 (0.0%)	1.00	0.71

Response scale: 1 = totally agree; 2 = partially agree; 3 = neutral; 4 = partially disagree; 5 = totally disagree. I-CVI, item-level Content Validity Index, calculated as the proportion of ratings 1 or 2. Alpha values in the final column are standardized alpha if the item was deleted.

In the structure and presentation domain, total agreement ranged from 95.5% to 100.0%. All participants totally agreed that the text was interesting and that the suggested content was appropriate. Font size, illustrations, clarity, and *cordel*-style writing also received favorable ratings from all participants (Table 2).

In the relevance domain, total agreement ranged from 86.4% to 95.5%. All participants provided favorable ratings for the coverage of necessary topics, usefulness for home care, support for reflection and behavioral change, and relevance for pregnant women with diabetes (Table 2).

Because all ratings were either total or partial agreement, every target-population item achieved an I-CVI of 1.00 and the S-CVI/Ave was 1.00 (Table 2).

### Internal consistency of target-population responses

Across the 14 target-population items, raw Cronbach’s alpha was 0.67 and standardized alpha was 0.66, indicating modest internal consistency in this small exploratory sample. The mean inter-item correlation was 0.14 (Tables 2 and 3).

**Table 3:** Overall internal-consistency parameters for target-population evaluations of the educational *cordel* (n = 22), Fortaleza, Ceará, Brazil, 2024.

Metric	Value
Raw Cronbach’s alpha	0.67
Standardized Cronbach’s alpha	0.66
Mean inter-item correlation (R̄)	0.14

Standardized alpha if item deleted ranged from 0.53 to 0.71. Removal of the item concerning the relevance of the technology produced the highest value (0.71); the small changes across items did not identify a single item whose removal would substantially improve consistency (Table 2).

The combination of uniformly favorable I-CVIs and modest alpha suggests a ceiling effect and limited response variability. These indices describe different properties and should therefore be interpreted together rather than as interchangeable evidence.

Overall response-consistency estimates are summarized in Table 3.

### Characteristics of expert judges

The 40 expert judges were equally distributed between HGCC and MEAC. Their mean age was 47.2 years (SD 10.5), 36 (90.0%) were women, 39 (97.5%) were obstetric nurses, and one (2.5%) was an obstetrician (Table 4).

**Table 4:** Sociodemographic and professional characteristics of expert judges who evaluated the educational *cordel* (n = 40), Fortaleza, Ceará, Brazil, 2025.

Variable	n	%
**Institution of origin**		
HGCC	20	50.0
MEAC	20	50.0
**Age (years)**	47.2 ± 10.5	
**Sex**		
Female	36	90.0
Male	4	10.0
**Profession**		
Obstetric nurse	39	97.5
Obstetrician	1	2.5
**Time since graduation (years)**	17.0 (12.0–29.0)	
**Experience in women’s health care**		
≤ 2 years	1	2.5
2–5 years	2	5.0
5–10 years	7	17.5
> 10 years	30	75.0

Categorical variables are presented as n (%). Continuous variables are presented as mean ± standard deviation or median (interquartile range).

Median time since graduation was 17.0 years (IQR 12.0–29.0), and 30 judges (75.0%) reported more than 10 years of experience in women’s health care (Table 4).

#### Expert evaluation of the educational *cordel.*

In the objectives domain, total agreement ranged from 67.5% to 85.0%. When total and partial agreement were combined, favorable responses ranged from 95.0% to 100.0%. Two neutral ratings were recorded for language appropriateness, and one neutral rating each for alignment with target-population needs and consistency with Ministry of Health recommendations (Table 5).

**Table 5:** Expert ratings, item-level content validity, and alpha if item deleted for the educational *cordel* (n = 40), Fortaleza, Ceará, Brazil, 2025.

Item	Response scale, n (%)					I-CVI	Alpha if item deleted
	1	2	3	4	5		
**Objectives**							
Alignment with target population needs	32 (80.0%)	7 (17.5%)	1 (2.5%)	0 (0.0%)	0 (0.0%)	0.98	0.97
Appropriate language for the target population	27 (67.5%)	11 (27.5%)	2 (5.0%)	0 (0.0%)	0 (0.0%)	0.95	0.97
Provides information to support self-care practices	31 (77.5%)	9 (22.5%)	0 (0.0%)	0 (0.0%)	0 (0.0%)	1.00	0.96
Encourages behavioral changes for self-care	32 (80.0%)	8 (20.0%)	0 (0.0%)	0 (0.0%)	0 (0.0%)	1.00	0.96
Visual presentation captures audience interest	31 (77.5%)	9 (22.5%)	0 (0.0%)	0 (0.0%)	0 (0.0%)	1.00	0.96
Potential for scientific dissemination	30 (75.0%)	10 (25.0%)	0 (0.0%)	0 (0.0%)	0 (0.0%)	1.00	0.96
Alignment with institutional objectives	34 (85.0%)	6 (15.0%)	0 (0.0%)	0 (0.0%)	0 (0.0%)	1.00	0.96
Consistency with Ministry of Health recommendations	33 (82.5%)	6 (15.0%)	1 (2.5%)	0 (0.0%)	0 (0.0%)	0.98	0.96
**Structure and presentation**							
Technology appropriate for pregnant women	28 (70.0%)	11 (27.5%)	1 (2.5%)	0 (0.0%)	0 (0.0%)	0.98	0.96
Messages are clear and direct	27 (67.5%)	13 (32.5%)	0 (0.0%)	0 (0.0%)	0 (0.0%)	1.00	0.96
Information is scientifically accurate	29 (72.5%)	11 (27.5%)	0 (0.0%)	0 (0.0%)	0 (0.0%)	1.00	0.96
Cover is attractive and representative	34 (85.0%)	6 (15.0%)	0 (0.0%)	0 (0.0%)	0 (0.0%)	1.00	0.96
Title and text size are appropriate	30 (75.0%)	10 (25.0%)	0 (0.0%)	0 (0.0%)	0 (0.0%)	1.00	0.96
Cordel format facilitates understanding	32 (80.0%)	8 (20.0%)	0 (0.0%)	0 (0.0%)	0 (0.0%)	1.00	0.96
Logical organization of content	29 (72.5%)	10 (25.0%)	1 (2.5%)	0 (0.0%)	0 (0.0%)	0.98	0.96
Font size is appropriate	29 (72.5%)	10 (25.0%)	1 (2.5%)	0 (0.0%)	0 (0.0%)	0.98	0.96
Illustrations support the content	29 (72.5%)	11 (27.5%)	0 (0.0%)	0 (0.0%)	0 (0.0%)	1.00	0.96
Amount of information is appropriate	30 (76.9%)	9 (23.1%)	0 (0.0%)	0 (0.0%)	0 (0.0%)	1.00	0.96
**Relevance**							
Covers essential content on gestational diabetes	30 (75.0%)	10 (25.0%)	0 (0.0%)	0 (0.0%)	0 (0.0%)	1.00	0.96
Supports nurses in home-based guidance	31 (77.5%)	9 (22.5%)	0 (0.0%)	0 (0.0%)	0 (0.0%)	1.00	0.96
Enables post-discharge follow-up	25 (62.5%)	13 (32.5%)	2 (5.0%)	0 (0.0%)	0 (0.0%)	0.95	0.97
Aligned with Ministry of Health care guidelines	28 (70.0%)	11 (27.5%)	1 (2.5%)	0 (0.0%)	0 (0.0%)	0.98	0.96
Promotes behavioral change	28 (70.0%)	12 (30.0%)	0 (0.0%)	0 (0.0%)	0 (0.0%)	1.00	0.96
Suitable as a discharge guidance tool	27 (67.5%)	12 (30.0%)	1 (2.5%)	0 (0.0%)	0 (0.0%)	0.98	0.96
Technology is relevant for pregnant women with diabetes	31 (79.5%)	8 (20.5%)	0 (0.0%)	0 (0.0%)	0 (0.0%)	1.00	0.96

Response scale: 1 = totally agree; 2 = partially agree; 3 = neutral; 4 = partially disagree; 5 = totally disagree. I-CVI, item-level Content Validity Index, calculated as the proportion of ratings 1 or 2 among valid responses. Alpha values in the final column are standardized alpha if the item was deleted. One response was missing for ‘Amount of information is appropriate’ and ‘Technology is relevant for pregnant women with diabetes.’ Overall raw alpha = 0.96; standardized alpha = 0.97; mean inter-item correlation = 0.53; Kendall’s W = 0.039.

In the structure and presentation domain, total agreement ranged from 67.5% to 85.0%, while favorable responses ranged from 97.5% to 100.0%. The cover received the highest total agreement (85.0%). One response was missing for the item on amount of information (Table 5).

In the relevance domain, total agreement ranged from 62.5% to 79.5%, and favorable responses ranged from 95.0% to 100.0%. The item on post-discharge follow-up had the lowest total agreement (62.5%) but 95.0% favorable agreement after partial agreement was included. One response was missing for the final relevance item (Table 5).

### Content validity, internal consistency, and concordance among experts

Expert I-CVIs ranged from 0.95 to 1.00, exceeding the prespecified 0.80 threshold for every item. The S-CVI/Ave was 0.99 overall (0.99 for objectives, 0.99 for structure and presentation, and 0.99 for relevance after rounding). Raw Cronbach’s alpha was 0.96, standardized alpha was 0.97, and the mean inter-item correlation was 0.53. Standardized alpha if item deleted ranged from 0.96 to 0.97 (Table 5).

Kendall’s W was 0.039, indicating weak concordance in the relative ordering of item ratings. This result coexisted with high absolute favorable agreement and should be interpreted in the context of the restricted response range and ceiling effects. Alpha, I-CVI, and Kendall’s W address different measurement properties; a high alpha does not substitute for inter-rater concordance.

## Discussion

This study developed a culturally grounded *cordel* and found strong preliminary evidence of content validity and acceptability among hospitalized pregnant women with diabetes and expert judges. All target-population items were favorably rated, expert I-CVIs ranged from 0.95 to 1.00, and the overall S-CVI/Ave was 0.99. However, modest alpha in the target population and weak Kendall concordance among experts require cautious interpretation and do not establish effectiveness.

*Cordel* combines oral tradition, rhyme, narrative structure, and visual symbolism. Previous studies have described its use for communicating health information about HIV prevention, breastfeeding, and dengue, highlighting accessible language and correspondence with regional cultural practices [22–24]. Related work on digital storytelling and mobile health has likewise examined accessible narrative and multimodal education in chronic disease and maternal health [26–29]. The present study extends this approach to diabetes in pregnancy and explicitly links hospital discharge guidance with continued self-care.

Diabetes in pregnancy requires women to understand and act on multiple recommendations during a period of clinical and emotional vulnerability. Educational technologies can support, but not replace, individualized counseling, shared decision-making, and coordinated care between maternity services and primary care [20,30,31].

The uniformly favorable target-population ratings suggest that participants considered the material understandable, appropriately presented, and relevant. Nevertheless, these results reflect perceived adequacy immediately after exposure; they do not demonstrate gains in knowledge, sustained behavior change, glycemic control, or continuity of care. The absence of negative ratings also produced limited variability, which helps explain why alpha remained below 0.70 despite I-CVIs of 1.00.

Among experts, high I-CVIs support the adequacy of the content, structure, and relevance domains. The high alpha indicates consistency across item responses, whereas Kendall’s W indicates little concordance in how judges ranked items relative to one another. Reporting both measures prevents a high alpha from being misinterpreted as evidence of strong inter-rater agreement.

Culturally grounded communication is especially relevant where technical language, low health literacy, and social inequities can limit engagement with conventional materials. By translating recommendations into familiar language and rhythm, *cordel* may facilitate dialogue between women, families, and health professionals [20,22–24]. The illustrated booklet broadens the modes through which discharge guidance can be discussed with participants, although its usability and accessibility were not formally evaluated in this study.

Hospital discharge is a vulnerable transition in which incomplete communication can undermine adherence and follow-up. A portable educational resource may reinforce guidance on diet, medication, glucose monitoring, warning signs, postpartum care, and return to primary care. Its implementation should be integrated with referral pathways and direct communication between maternity and primary care teams [31–34].

The *cordel* may therefore serve as a low-cost adjunct to nursing and interprofessional education in hospital and primary care settings. Its contribution lies in supporting understandable, culturally responsive conversations rather than functioning as a stand-alone intervention or substitute for clinical follow-up.

The findings also underscore the distinction between validating educational content and demonstrating clinical effectiveness. This study assessed ratings of objectives, presentation, and relevance; it did not test whether the material changed self-care behavior or clinical outcomes.

The target-population alpha of 0.67 should be viewed as exploratory. Alpha is influenced by sample size, number of items, response variability, and the dimensionality of the evaluation form. Accordingly, the value neither invalidates the favorable content ratings nor supports claims of established psychometric reliability.

### Limitations and future directions

The convenience sample was small and drawn from two tertiary hospitals in one Brazilian city, limiting transferability to other regions, outpatient settings, and populations with different cultural or literacy profiles. No a priori sample-size calculation was performed. The two pilot participants were retained in the final target-population sample after minor revisions, which may have introduced familiarity or incorporation bias.

The study examined one culturally specific version of the material. Its use in other regions or countries will require cultural adaptation and renewed evaluation rather than assuming that validation can be transferred unchanged.

The structured evaluation form was adapted for this study, and evidence for its construct validity was not established. Internal consistency and Kendall’s W were based on a restricted response range with pronounced ceiling effects. Test-retest reliability, responsiveness, readability, and construct validity were not assessed.

The Patient Education Materials Assessment Tool was not used; consequently, understandability and actionability were inferred from study-specific items rather than measured with a standardized instrument [35]. The usability and accessibility of the illustrated booklet were not evaluated with standardized instruments.

Future multicenter studies should recruit larger and more diverse samples; use standardized measures of understandability, actionability, readability, and usability; separate pilot and validation samples; and examine test-retest performance. Pragmatic or controlled studies should then evaluate whether the *cordel* improves knowledge, self-care behaviors, glycemic management, postpartum follow-up, and continuity between hospital and primary care.

## Conclusion

The educational *cordel* showed strong preliminary content validity and high acceptability among hospitalized pregnant women with GDM or pre-existing T2DM and among expert judges. Its culturally grounded narrative and visual format may support discharge education and continuity of self-care. These findings justify broader validation and effectiveness testing but do not yet establish psychometric robustness or clinical benefit.

## Supporting information

S1 File Final educational cordel in Portuguese. (PDF)

S2 File Final educational cordel in English. (PDF)

S3 File English-language target-population and expert-judge evaluation forms for the educational cordel. (DOCX)
