# Supplementary material for: Culturally grounded health communication to support self-care among pregnant women with diabetes: Development and content validation of an educational cordel in Brazil
Source: PLoS One. 2026 Sep 11;21(9):e0357959. doi: 10.1371/journal.pone.0357959 (PMC13567755; doi:10.1371/journal.pone.0357959)
Supplement: Supplementary file 1 [file pone.0357959.s001.pdf]

DIABETES NA GESTAÇÃO REQUER TOTAL ATENÇÃO!

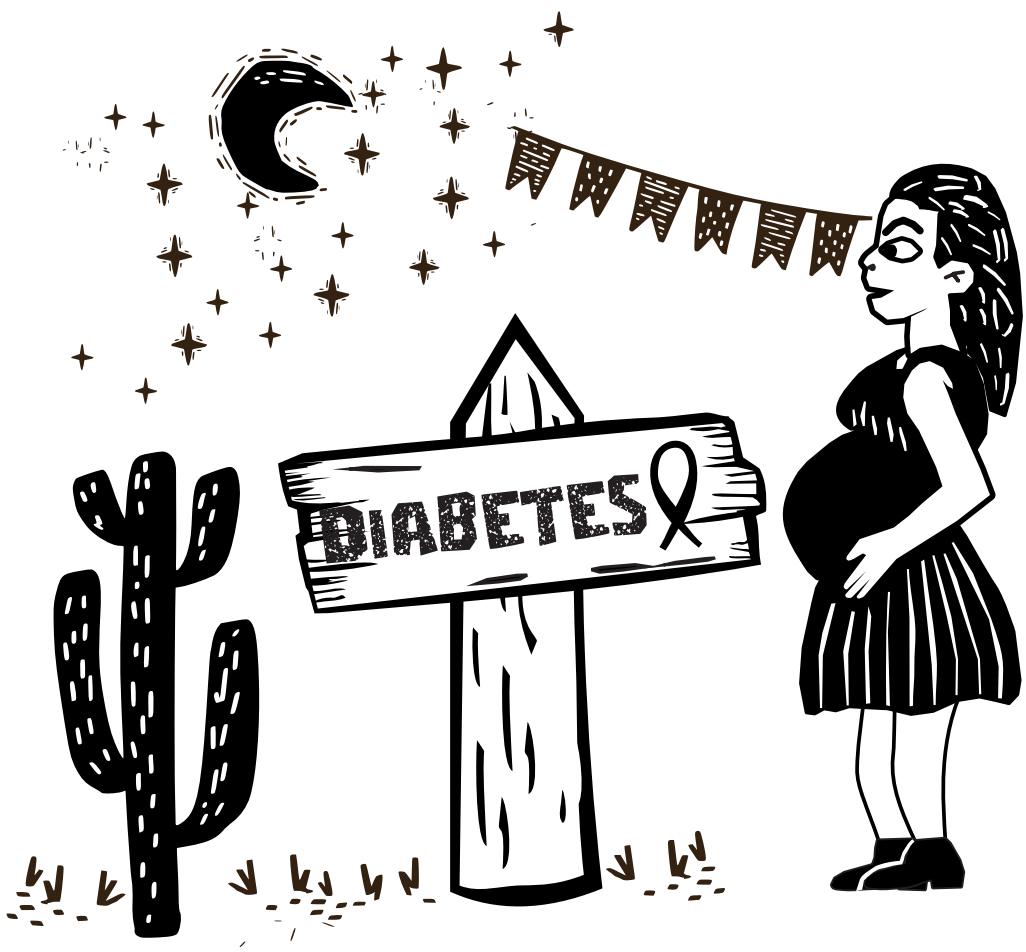

O que é mesmo diabetes?
Foi bom você perguntar
para tirar suas dúvidas
vamos agora explicar
assim desde o surgimento
também qual o tratamento
e como se comportar.

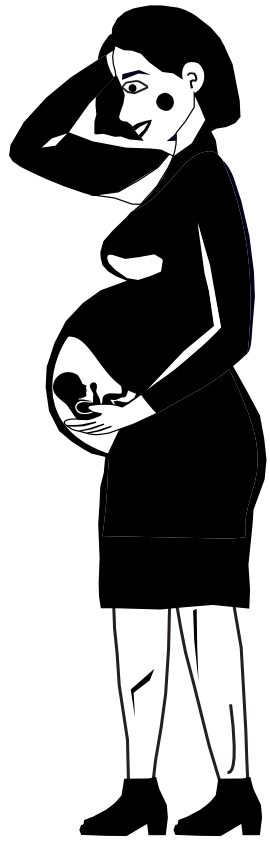

Diabetes, é alto nível
de glicose em sua veia
é dependência do açúcar
deixando a coisa bem feia
e o pâncreas sem insulina;
crie uma nova rotina
se não você se aperreia.

Quando o pâncreas não produz
insulina em quantidade
para transformar o açúcar
em força e vitalidade
surge então grande problema,
aí começa o seu dilema
passa a ser enfermidade.

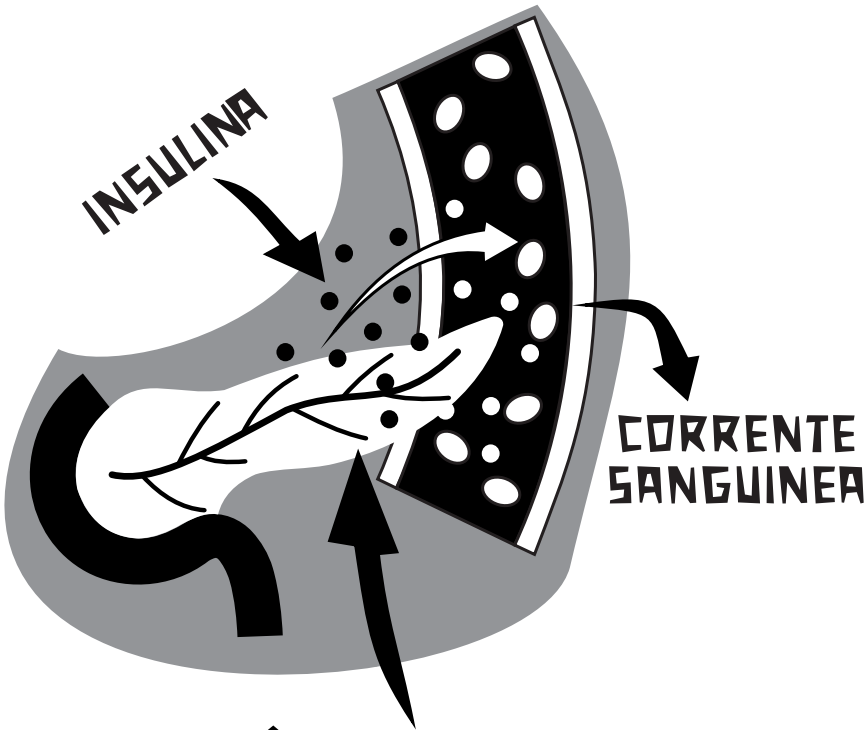

PÂNCREAS

Ao excesso dos açúcares
preste bastante atenção,
os ditos carboidratos,
são: massas, arroz e pão
tenha sempre disciplina
coma fruta e proteína
completando a refeição.

Açúcar que vem do leite,
chamado de lactose
mas temos o açúcar branco
conhecido sacarose
transforma-se em energia
talvez você não sabia
que no seu sangue, é glicose.

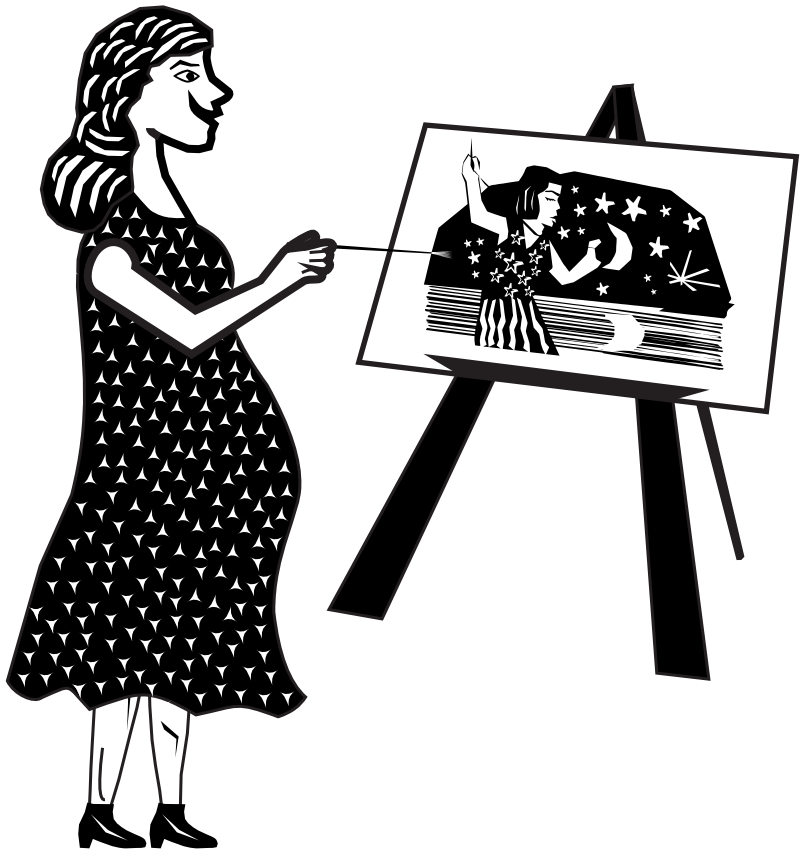

Com a glicose aumentada
motivo de precaução
a diabetes formada
temos preocupação,
medidas vamos tomar,
não vamos desesperar
pra tudo tem solução!

Mas não é contagiosa,
pode ser hereditária
criança, idoso, mulheres
e de qualquer faixa etária
não é nada interessante,
mais atenção à gestante
é atitude necessária.

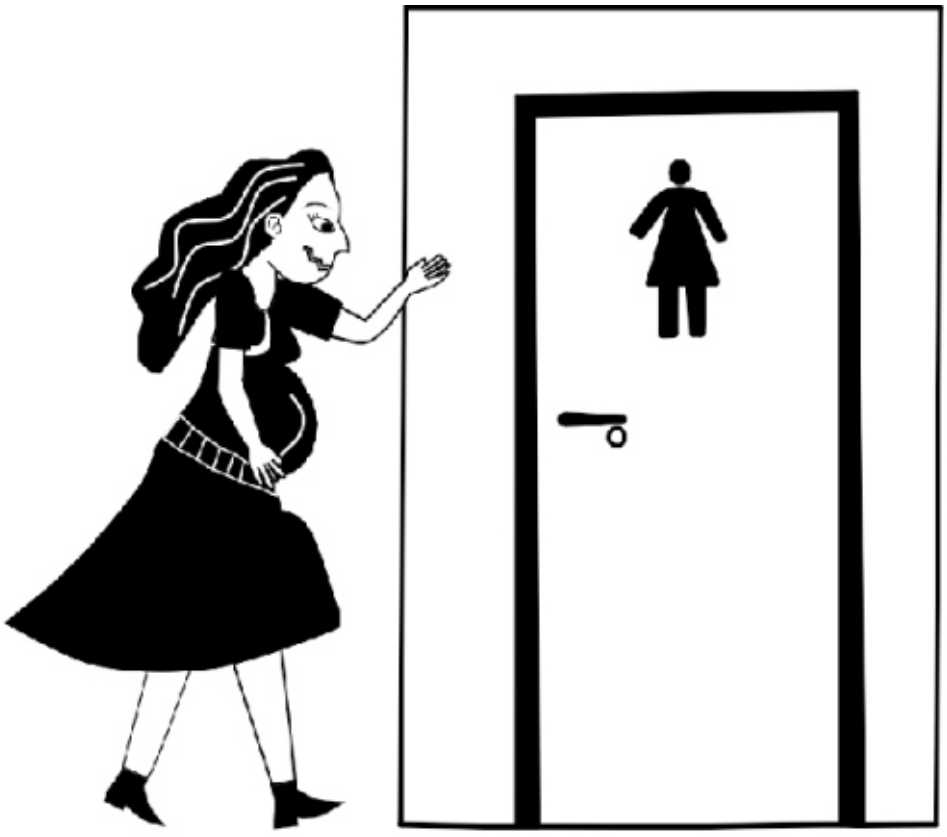

Fique atento aos sintomas
pra você não vacilar
com diabetes tipo 1
procure o peso observar
aquela fome constante
a sede que é incessante
e vontade de urinar.

Diabetes tipo 2
vem cheia de novidades
traz: fome, sede, fadiga
e muitas enfermidades,
na bexiga, pele e rins,
são todas muito ruins,
consta nas comorbidades.

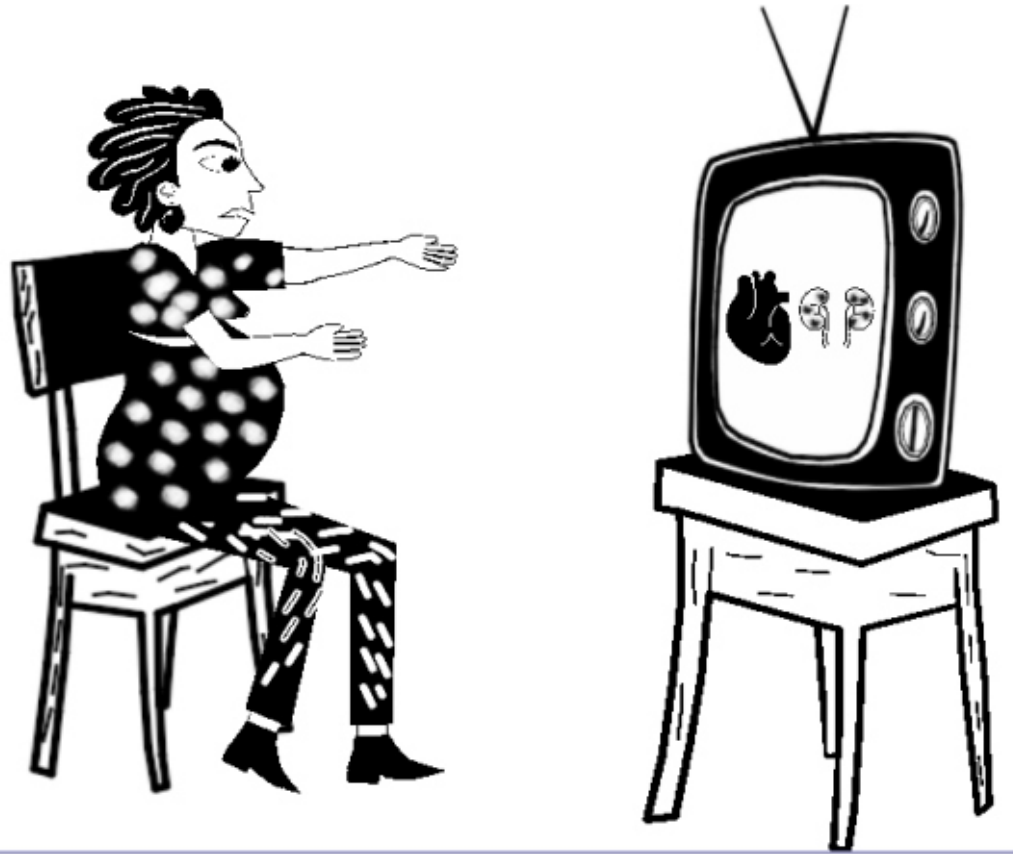

E durante a gravidez
ou seja no pré-natal
pesquise por diabetes
TIPO GESTACIONAL
sempre através dos exames
para não passar vexames
e ter um parto normal.

Gravidez eleva o risco
de surgir intolerância
ao açúcar e é preciso
manter uma vigilância,
pelo bem-estar fetal,
cuidados no pré-natal
que são de suma importância.

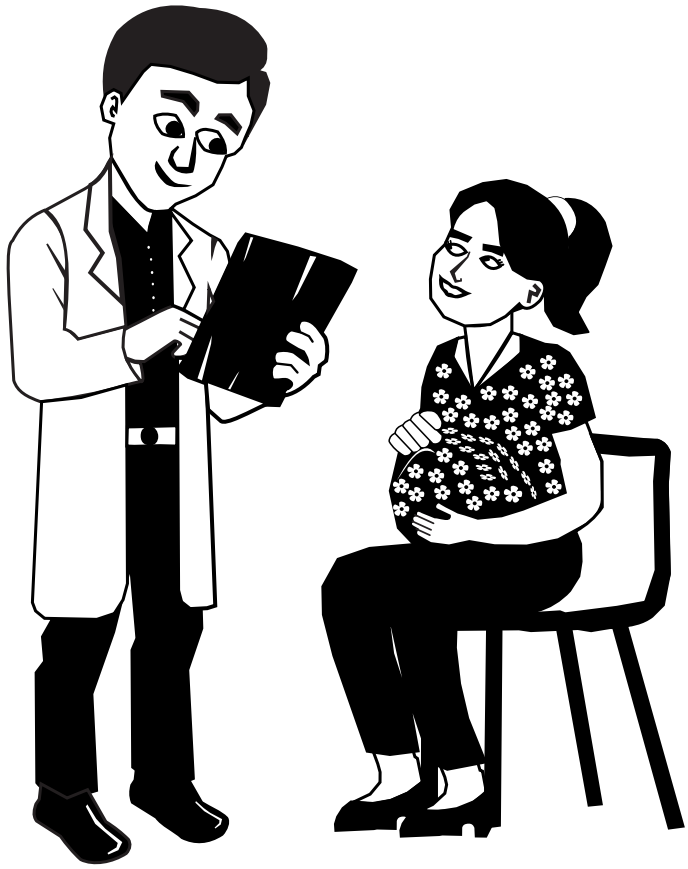

Ficar grávida, pretende?
Que seja então planejada.
evite o bebê doente
com glicose controlada
tome, uns três meses, folato
adéque o peso de fato
antes da vida gerada.

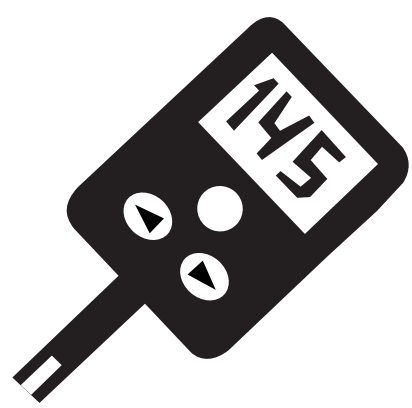

A glicose a gente mede
sempre em toda gestação
pois, seu doutor nunca esquece
ele dedica atenção
que é para num ter marmota
os valores ele anota
com carinho no cartão.

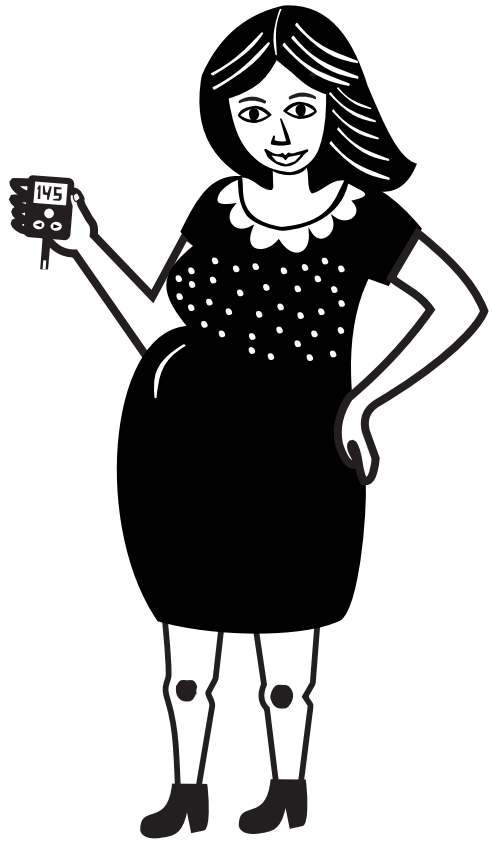

Grávida deve colher,
de jejum, a glicemia,
bem no início pra saber
se diabetes teria.
e se der normal, repete,
toma garapa de teste...
é assim que se vigia.

Durante o acompanhamento
é vigilância total
faça monitoramento
com fita pós prandial;
não precisa de ter medo
pois não dói furar o dedo
pra o bebê nascer normal!

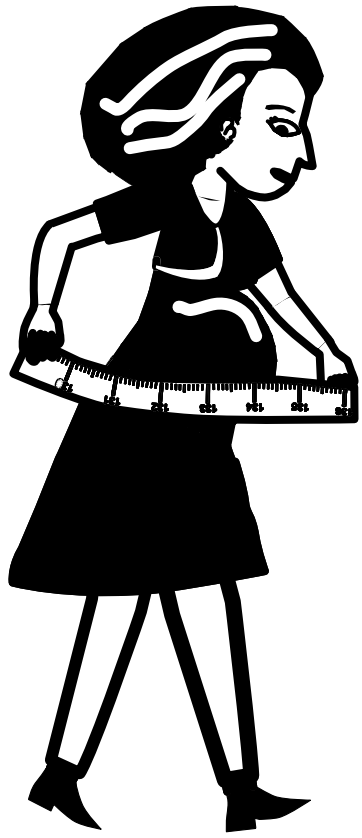

Enquanto a boca enche d'água
e de massas enche a pança
no ventre, o bebê se enxágua,
no fluido doce balança.
Aumenta tanto a barriga;
para dormir, uma briga
insônia dá comilança

Controlar taxa de açúcar,
tem que medir e anotar
pra hemorragia pós-parto
nas mãezinhas evitar
também parto prematuro
diabetes no futuro
pode a vida abreviar.

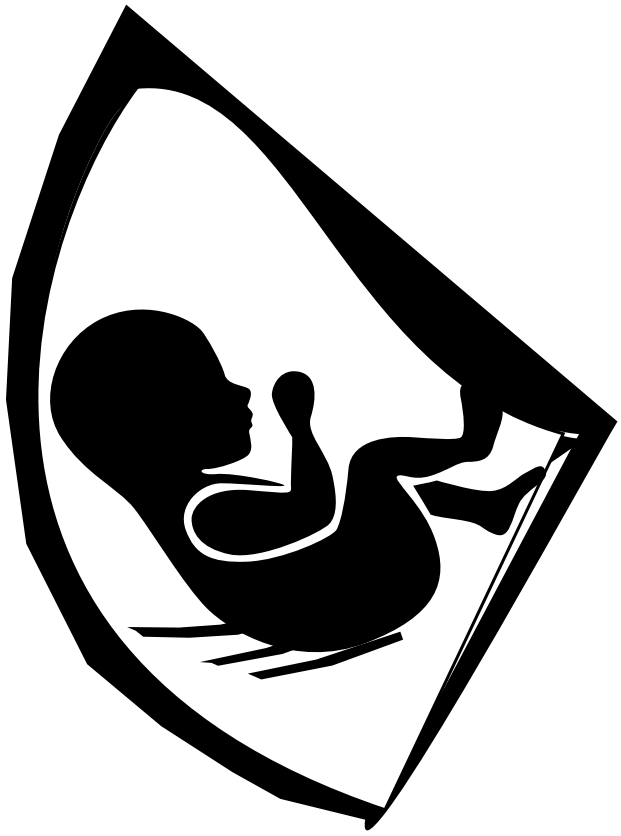

Pode muito interferir
caso tenha complicação
tal como hipoglicemia,
e dificil respiração,
nascer grande não é sorte
pode até levar à morte
ou nascer um bebezão.

Não devemos esquecer
de outros problemas falar
pois no esperado bebê
ela pode machucar
o nenê ficar grandão
problema no coração
corre o risco de causar!

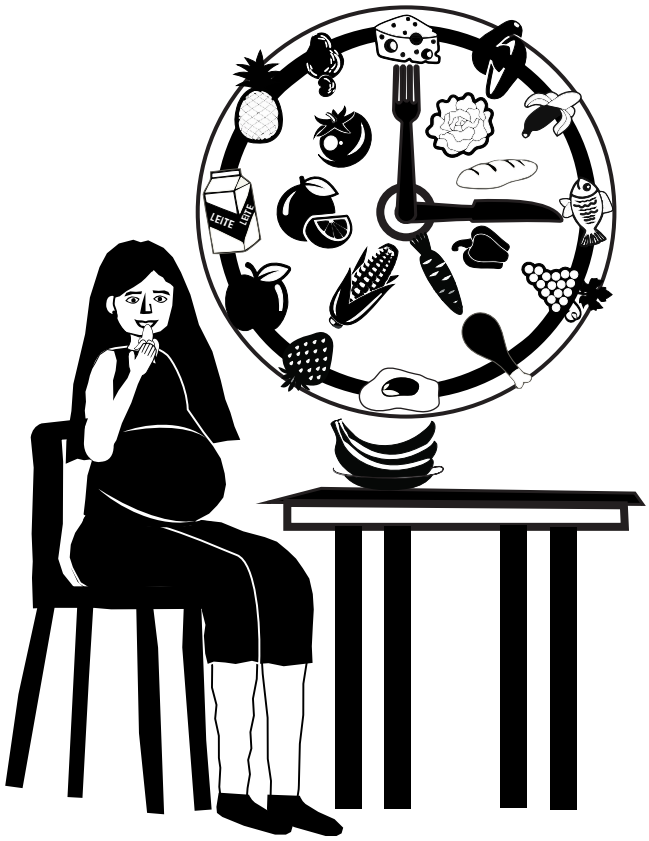

Para isto você precisa
manter sua disciplina,
horário de refeição
deve tornar-se rotina,
quantidades e medida
pra seguir normal a vida,
conforme se determina.

Ter seis refeições ao dia
é clara a orientação,
sem pressa, nem agonia
e boa mastigação.
Ao café, almoço e jantar,
três lanches acrescentar
sem exceder a porção!

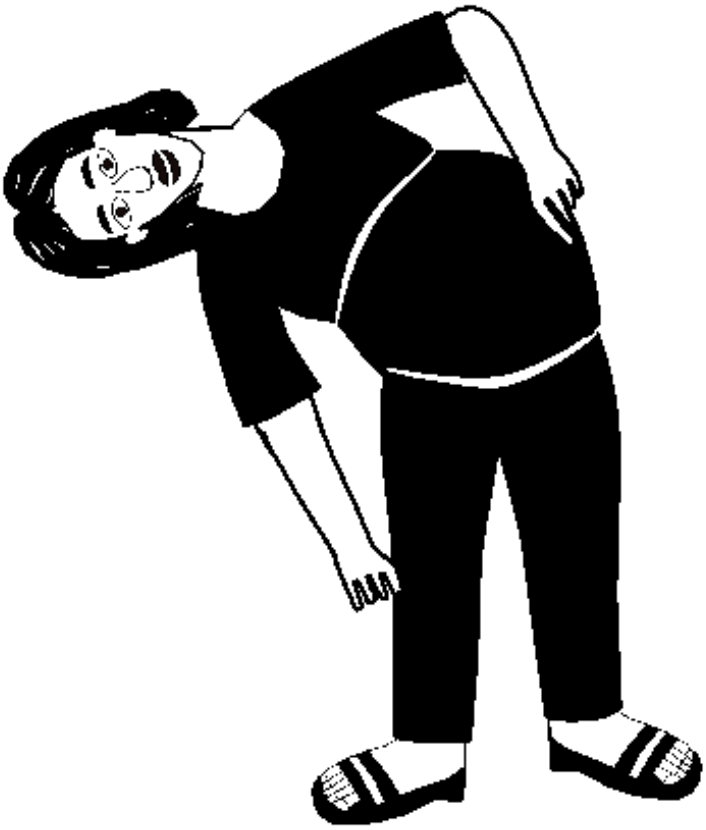

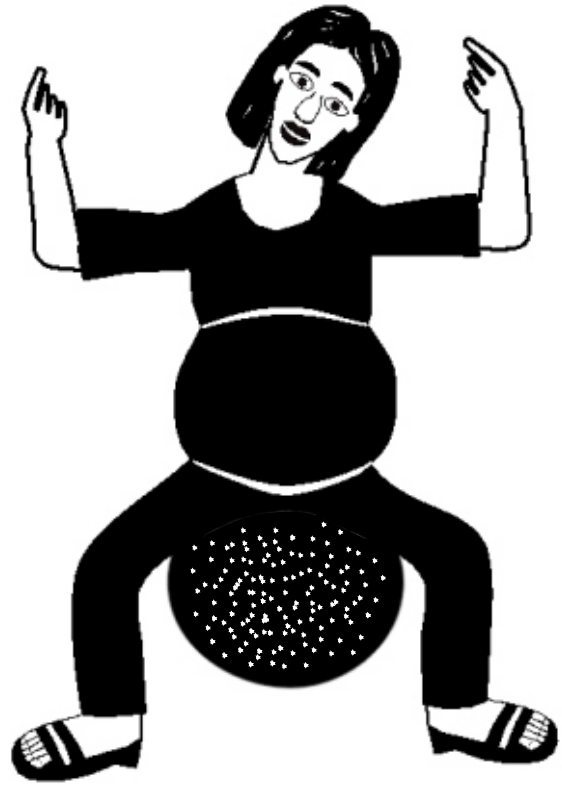

Mas pode e deve comer
uma rica refeição:
carnes, ovos, leite e frutas,
massa, arroz, milho e feijão,
verdura e oleaginosas
outras comidas gostosas,
siga a recomendação.

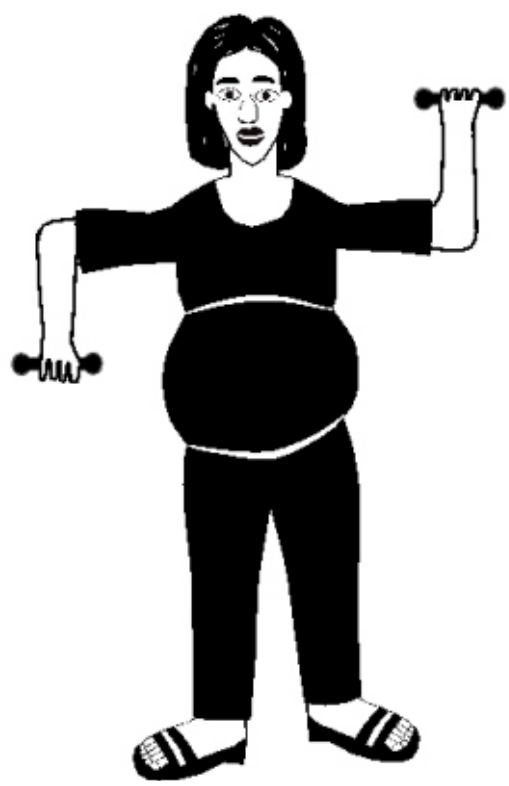

Algo não pode faltar
completando este processo
são exercícios físicos,
no combate ao excesso,
escolha o que lhe convém
porquê a lista contém
o segredo do sucesso.

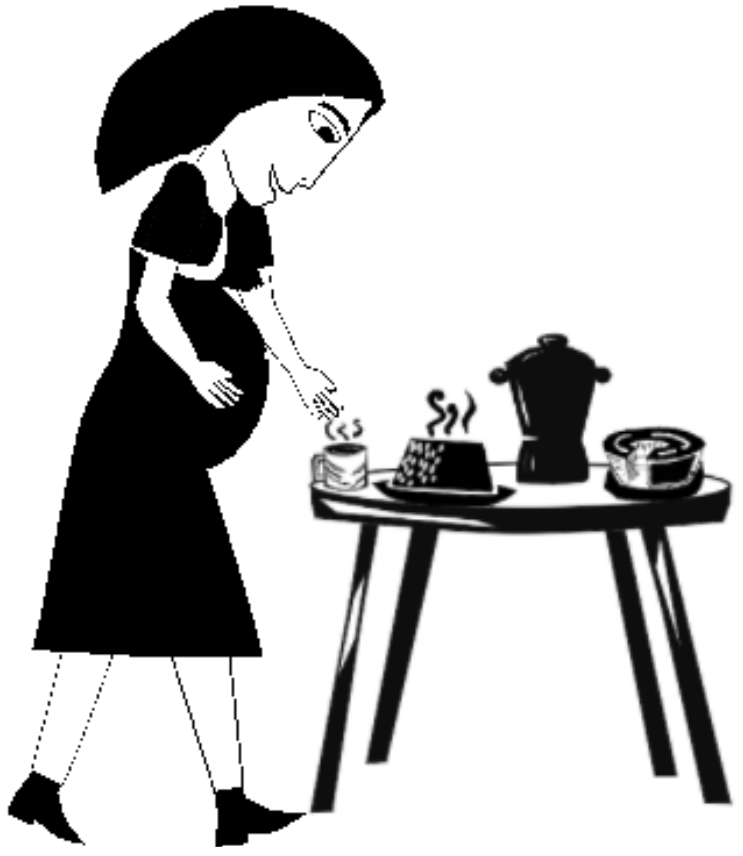

Diabética gestante,
veja a recomendação,
exercicio moderado:
caminhada, natação,
hidroginástica ou yoga,
o pilates tá em voga
e ajuda na gestação.

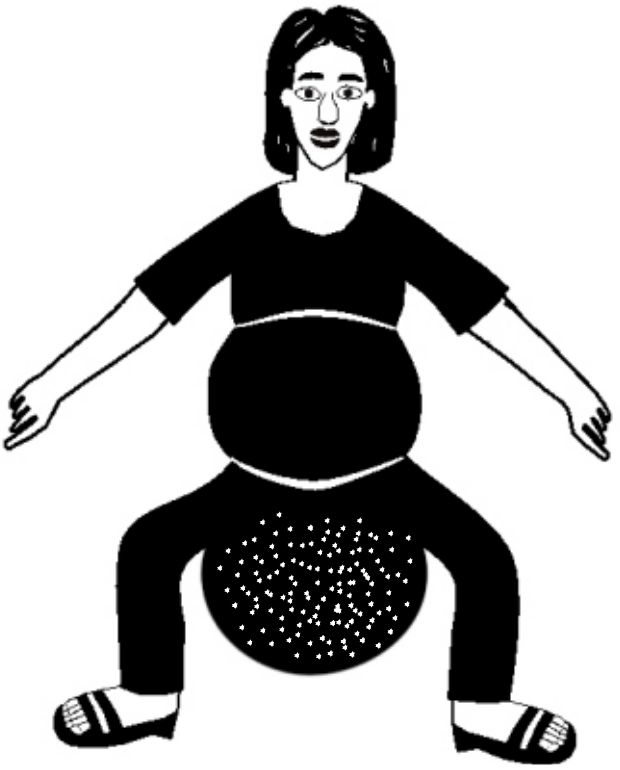

Prevenir, remediar
requer muito sacrificio
para manter o controle
tem que fazer exercício,
a frequente medição
seleta alimentação
garante mais beneficio.

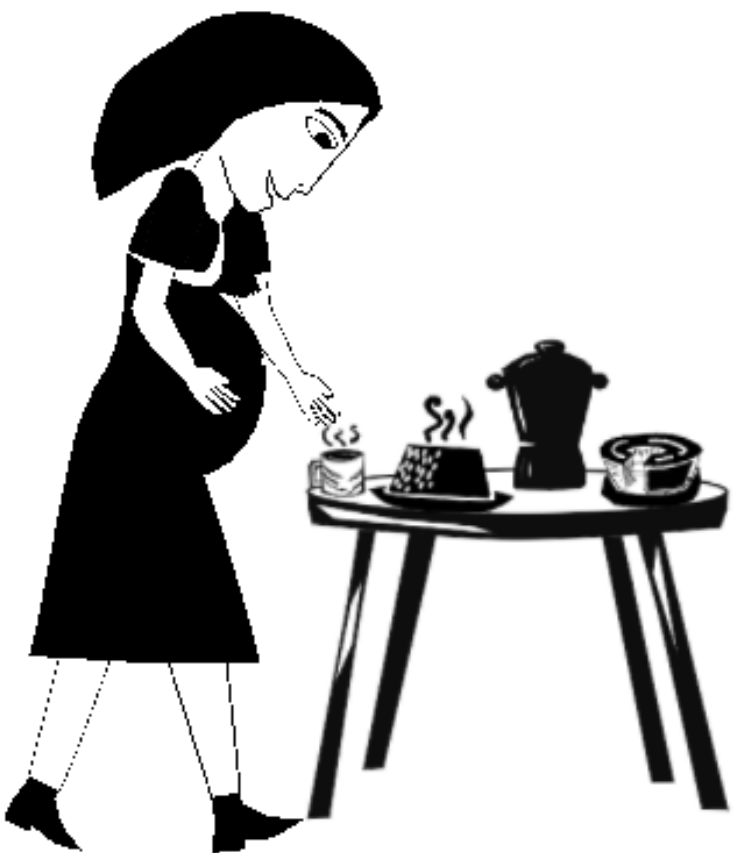

Se a vida é doce demais,
com diabetes complica,
deixe o açúcar para trás
massa faz mal, fica a dica!
E não coma a todo instante,
nem beba refrigerante;
senão, mais doente, fica.

Se a glicose não controla
bebê cresce exagerado
e pode vir antes da hora,
na UTI ser internado,
ser pesado e prematuro,
ser obeso no futuro,
ter parto mais demorado.
