# Supplementary material for: Culturally grounded health communication to support self-care among pregnant women with diabetes: Development and content validation of an educational cordel in Brazil
Source: PLoS One. 2026 Sep 11;21(9):e0357959. doi: 10.1371/journal.pone.0357959 (PMC13567755; doi:10.1371/journal.pone.0357959)
Supplement: Supplementary file 2 [file pone.0357959.s002.pdf]

DIABETES IN PREGNANCY REQUIRES YOUR FULL ATTENTION!

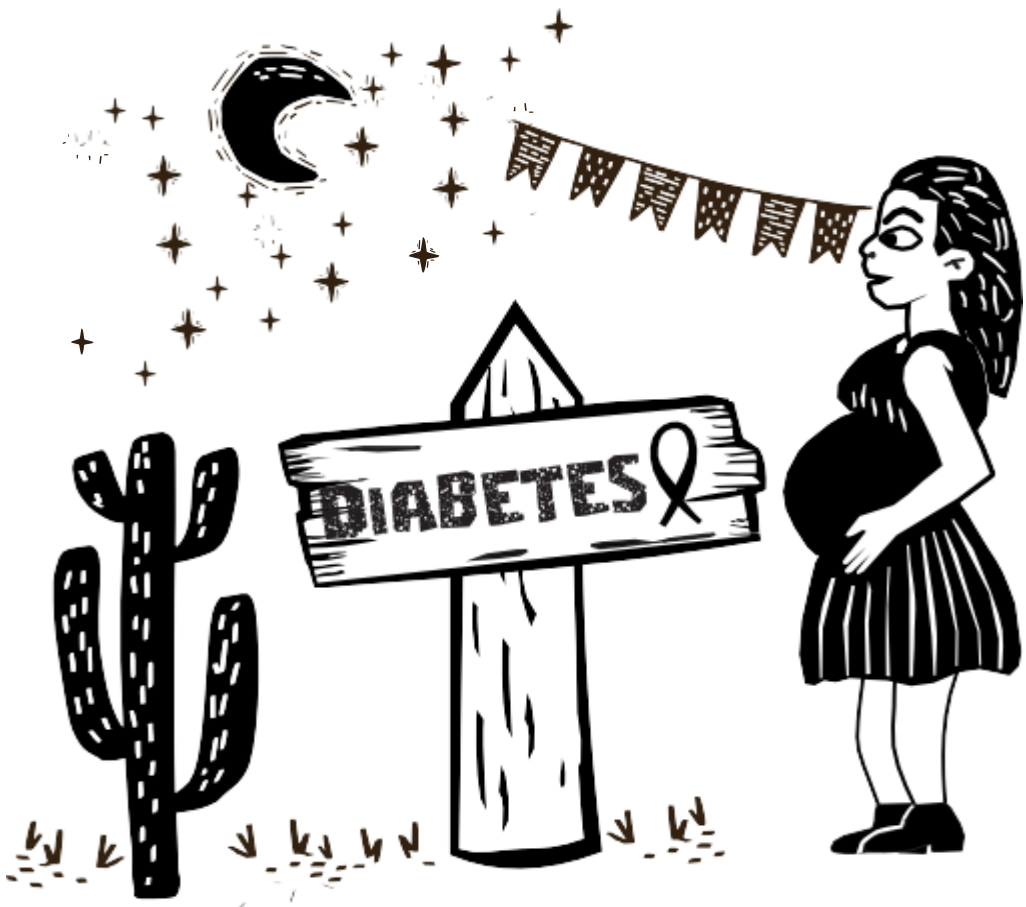

What exactly is diabetes? That is a good question to ask. Let us clear up your doubts: how it develops, how it is treated, and what you can do.

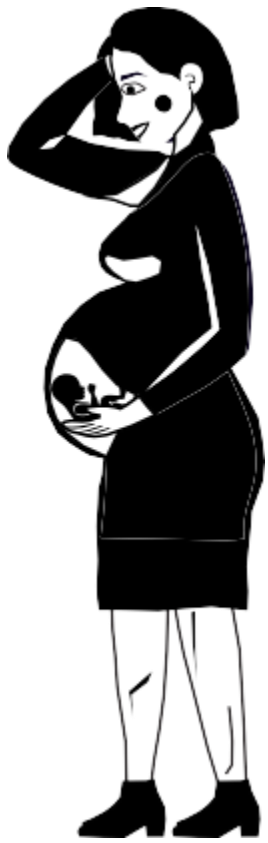

Diabetes means too much glucose flowing in your blood. When insulin is lacking, sugar builds up instead. A healthier daily routine can help you move ahead.

When the pancreas does not make enough insulin to turn glucose into energy and vitality, a serious problem begins—and diabetes becomes a reality.

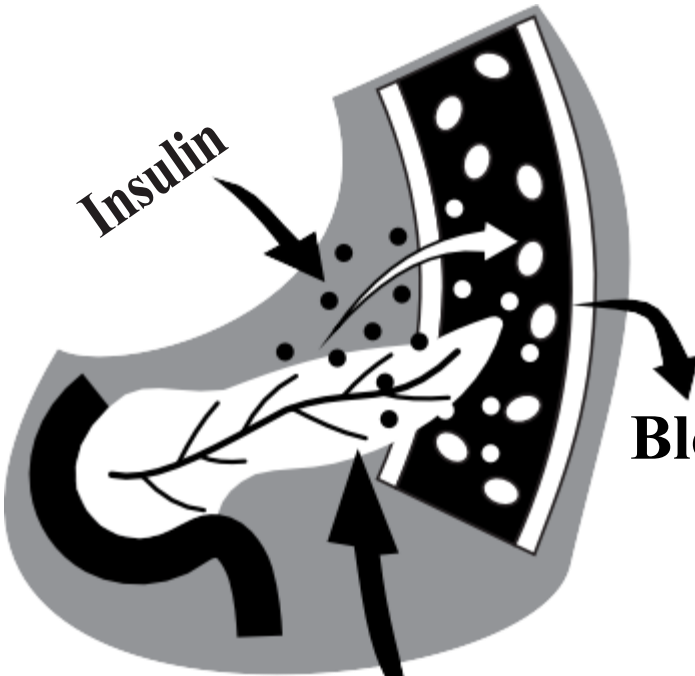

Pay close attention to excess sugar and carbohydrates such as pasta, rice, and bread. Keep portions balanced and add fruit and protein to every meal instead.

The sugar found in milk is called lactose; white table sugar is sucrose. Sugars are converted into energy, and in your bloodstream, they circulate as glucose.

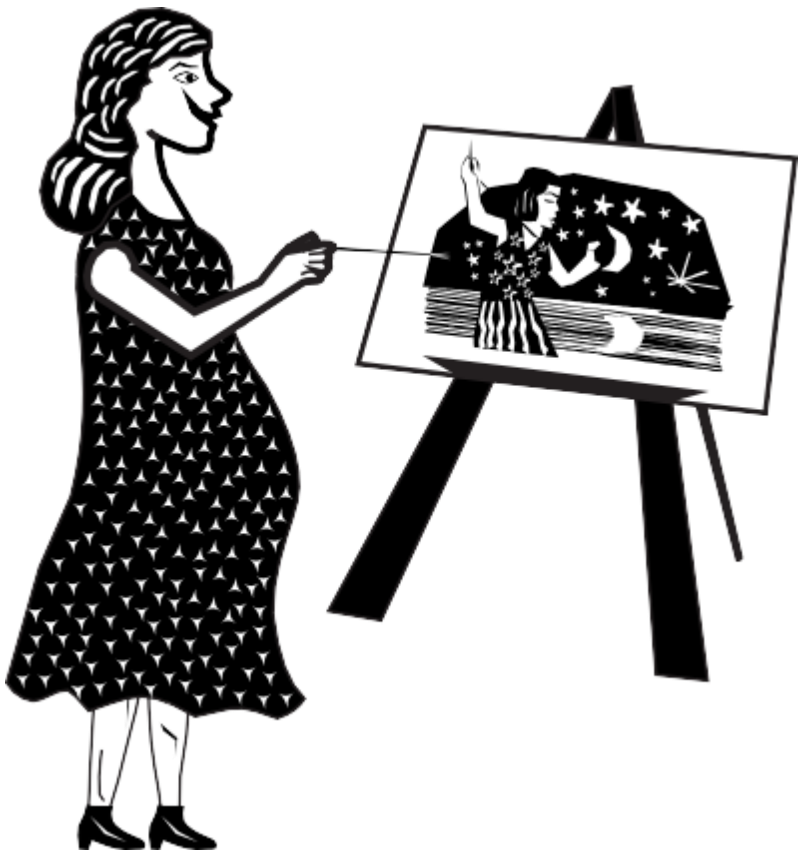

When blood glucose rises, caution is required. Diabetes is a concern, but action can be taken—do not despair, because proper care can provide a solution!

Diabetes is not contagious, although heredity may play a part. It can affect people of every age; during pregnancy, special attention is wise from the start.

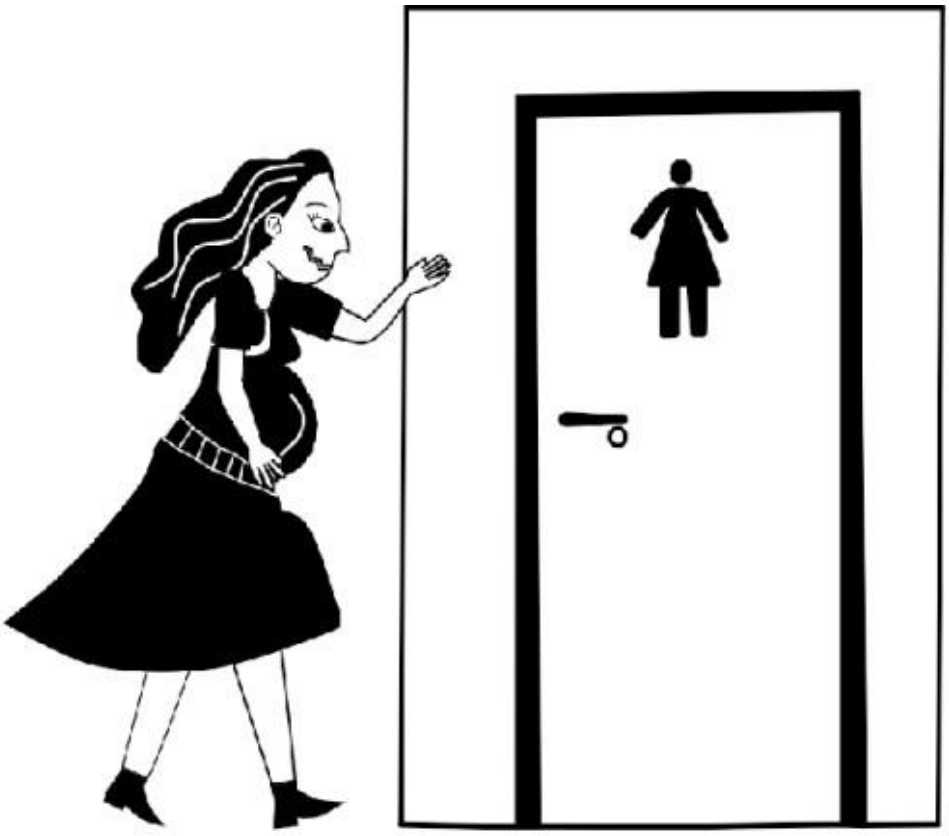

Watch for the warning signs of type 1 diabetes: unexplained weight loss, constant hunger, unrelenting thirst, and a frequent need to urinate.

Type 2 diabetes may bring hunger, thirst, and fatigue. It can also affect the bladder, skin, and kidneys and lead to other health conditions.

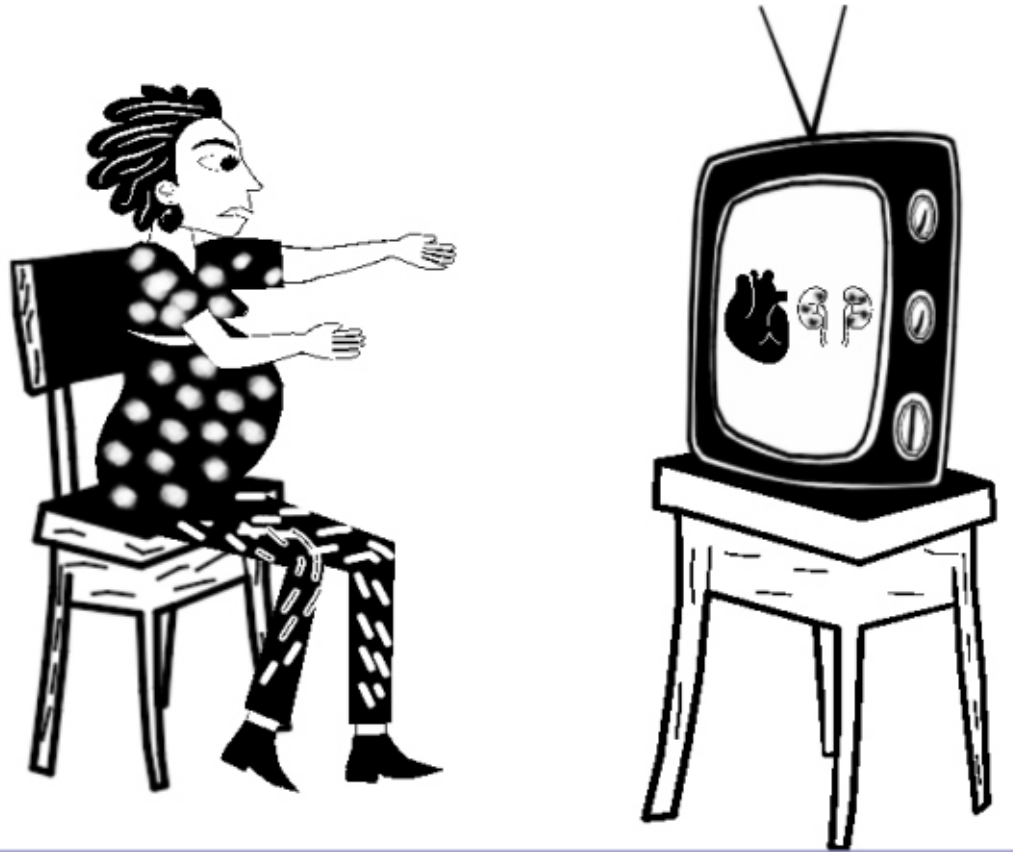

During pregnancy, prenatal care should include screening for GESTATIONAL DIABETES through the recommended tests, supporting a safer pregnancy and birth.

Pregnancy can increase the risk of glucose intolerance, so careful monitoring is essential. Prenatal care is of the utmost importance for the well-being of both mother and baby.

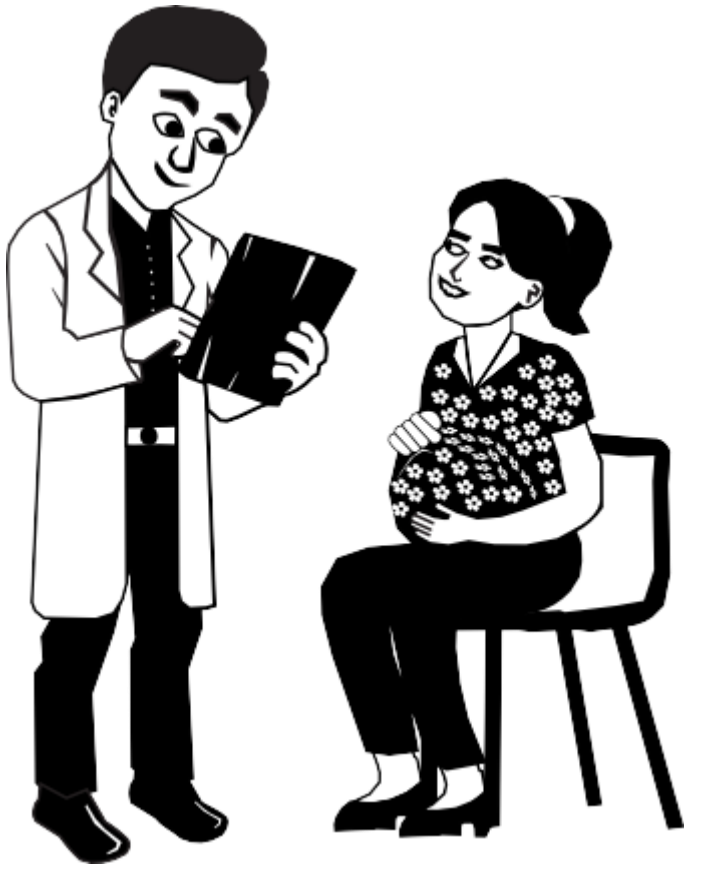

Planning to become pregnant? Prepare with care: control your glucose, aim for a healthy weight, and take folic acid as advised—ideally starting about three months beforehand.

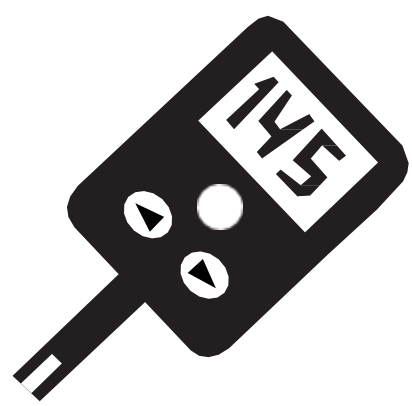

Glucose is checked throughout pregnancy. Your healthcare professional monitors the results carefully and records each value on your prenatal card.

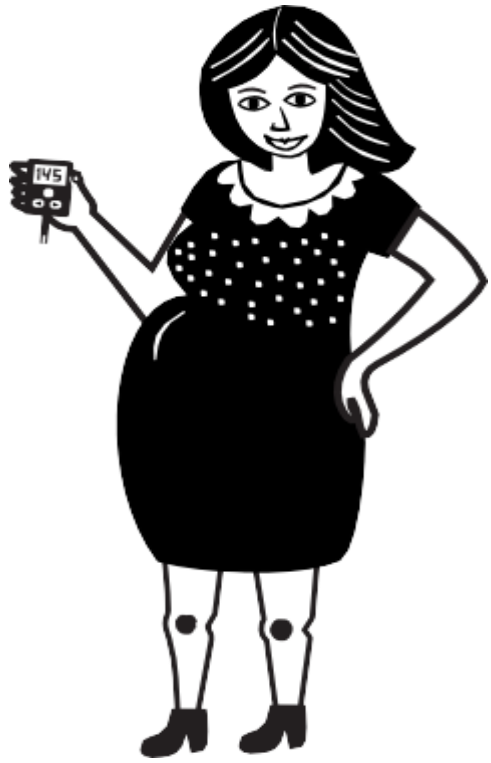

Early in pregnancy, fasting blood glucose should be tested. If the result is normal, an oral glucose tolerance test is performed later, as recommended.

Careful follow-up is essential. Check your post-meal glucose with a test strip when advised. A small finger prick helps protect you and supports your baby's healthy birth.

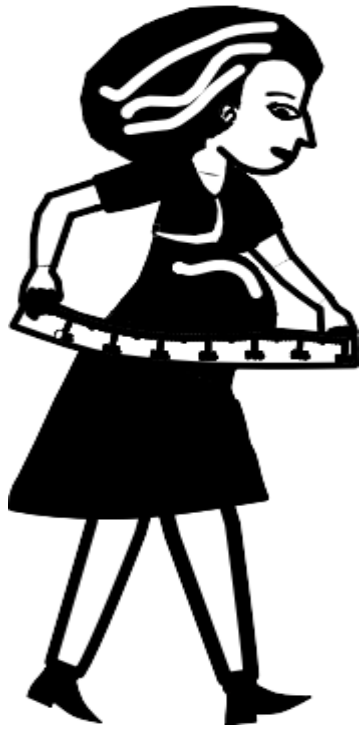

When cravings fill your plate
with sweets and refined
carbohydrates, glucose also
reaches the baby, who is
surrounded by amniotic fluid in
the womb.
As the belly grows, sleep
may become difficult—
and sleeplessness can
encourage overeating.

Measure and record your
glucose to reduce health
risks, including preterm
birth and complications
after delivery. Good
control also lowers future
risks for mother and child.

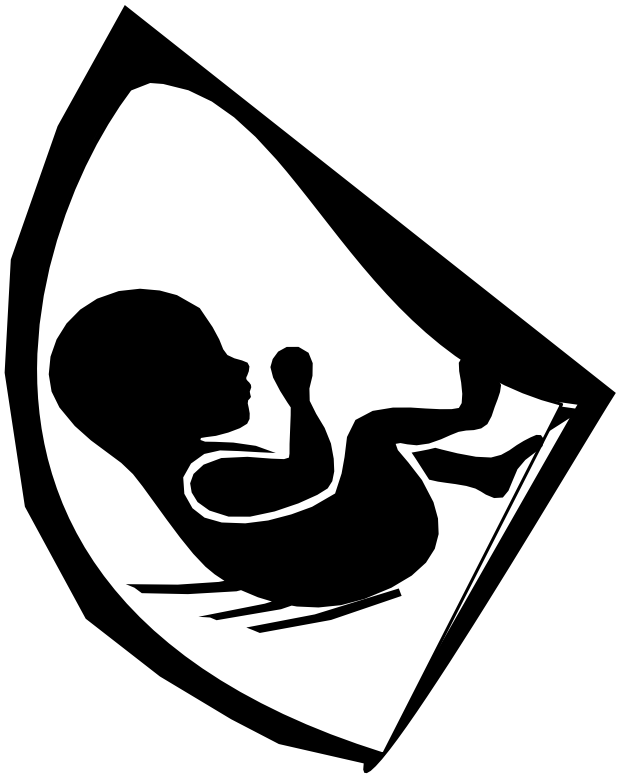

Complications may include low blood glucose in the newborn, breathing difficulties, and excessive birth weight. These conditions can be serious and require prompt care.

Other risks should not be forgotten: the baby may grow too large, birth injuries may occur, and the risk of heart problems may increase.

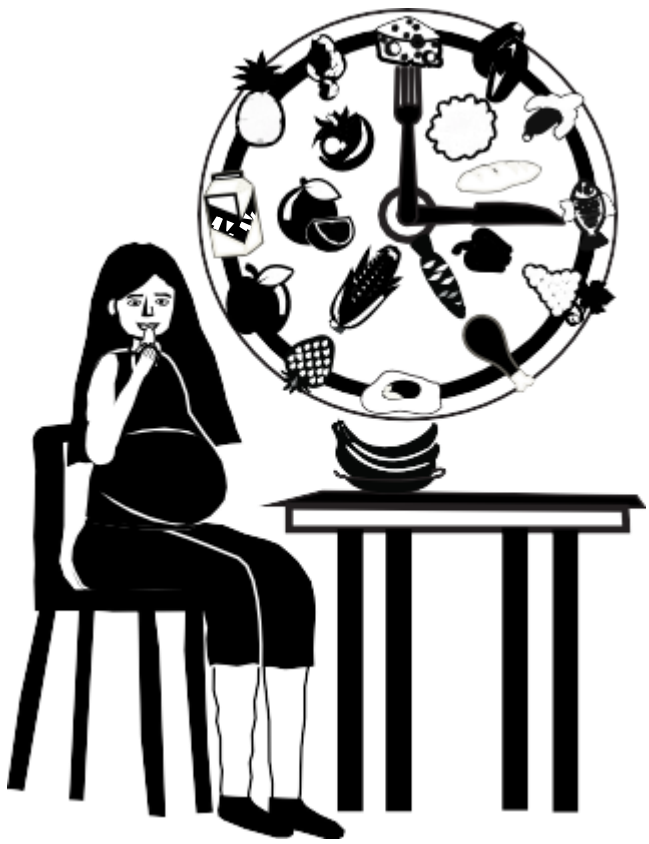

To care for yourself, keep a regular meal schedule and follow the recommended portions. Consistency helps you maintain a healthy daily routine.

If advised, have six small meals a day. Eat calmly, without rushing, and chew each bite well. Along with breakfast, lunch, and dinner, add three snacks without exceeding the recommended portions!

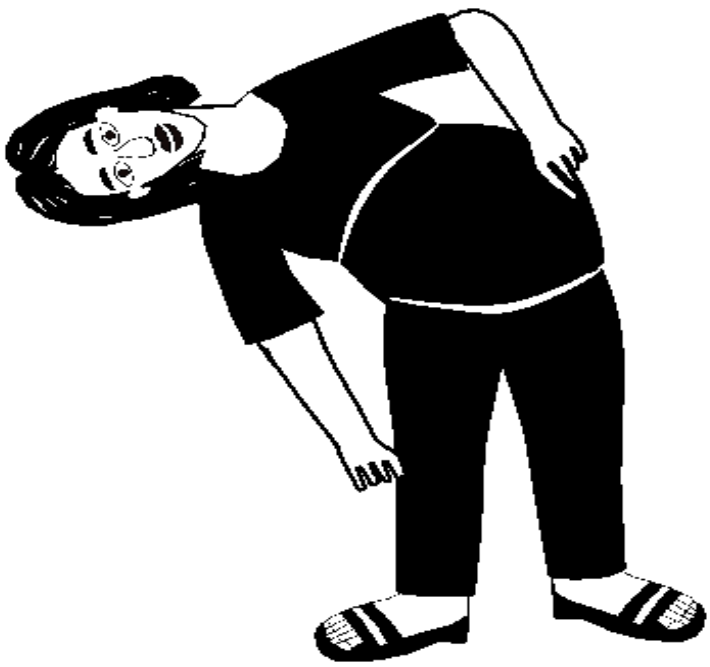

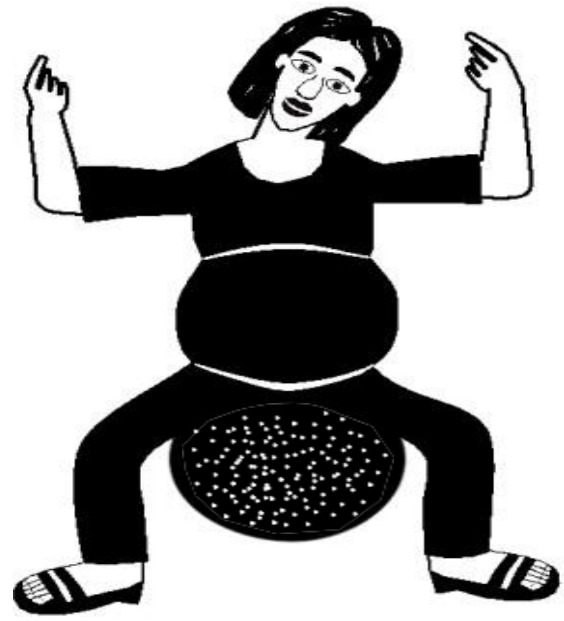

You can and should enjoy balanced meals: meat, eggs, milk, fruit, pasta, rice, corn, beans, vegetables, and nuts. Follow professional guidance and choose foods in suitable portions.

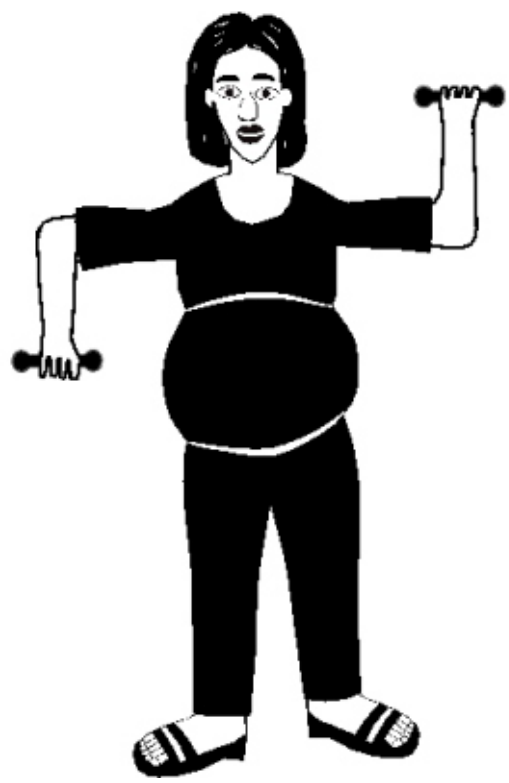

Physical activity is another important part of care. With professional guidance, choose an activity that suits you—regular movement can support successful control.

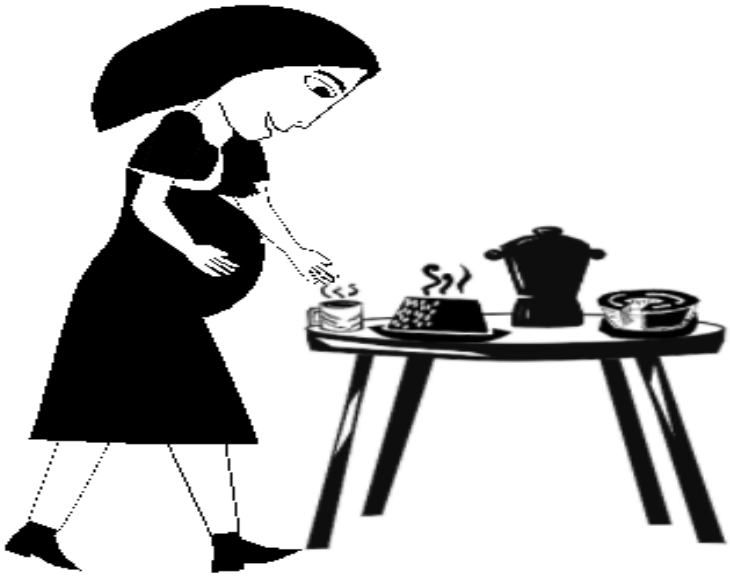

For pregnant women with diabetes, moderate activity may be recommended: walking, swimming, water aerobics, yoga, or Pilates can support well-being during pregnancy.

Prevention and treatment require commitment. Regular activity, frequent glucose checks, and carefully selected foods bring greater benefits and improve control.

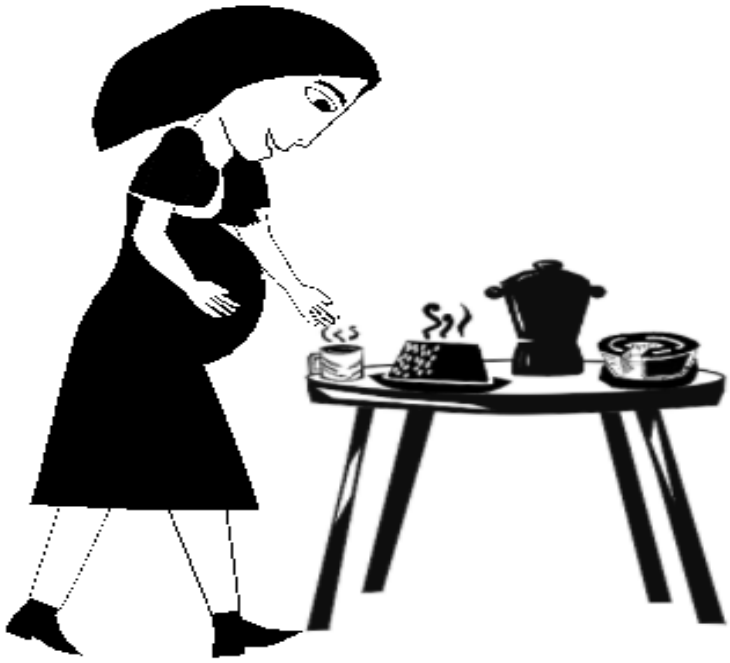

Too much sweetness complicates diabetes. Limit added sugar and refined carbohydrates, avoid constant snacking and sugary drinks, and follow your care team's advice.

If glucose is not controlled, the baby may grow too large or arrive too early, need neonatal intensive care, face a difficult delivery, and have a higher risk of obesity later in life.
