# Supplementary material for: Culturally grounded health communication to support self-care among pregnant women with diabetes: Development and content validation of an educational cordel in Brazil
Source: PLoS One. 2026 Sep 11;21(9):e0357959. doi: 10.1371/journal.pone.0357959 (PMC13567755; doi:10.1371/journal.pone.0357959)
Supplement: Supplementary file 3 [file pone.0357959.s003.docx]

**S2 FILE**

**EVALUATION OF THE EDUCATIONAL CORDEL ON DIABETES DURING PREGNANCY — TARGET POPULATION**

Participant code: ______________________________

Date: **/**/________

Institution/site: ______________________________

Interviewer/evaluator: ______________________________

PARTICIPANT CHARACTERISTICS

Referring hospital: ☐ HGCC ☐ MEAC

Age: __________ years

Self-reported race/skin color:

☐ White ☐ Black ☐ Brown ☐ Asian ☐ Indigenous ☐ Other

Occupation:

☐ Homemaker ☐ Other: __________________________________________

Marital status:

☐ Single ☐ Married ☐ Stable union ☐ Other: ______________________

Education level:

☐ Primary education ☐ Secondary education ☐ Higher education

Municipality of residence:

☐ Fortaleza ☐ Other: ___________________________________________

Monthly household income: BRL ____________________

Receives social benefits:

☐ No ☐ Yes. Which? ____________________________________________

Number of pregnancies: __________

Prenatal care start date: **/**/________

Gestational age at initiation: __________ weeks

Site where prenatal care began:

☐ HGCC ☐ MEAC ☐ Polyclinic ☐ Health center ☐ PHC unit ☐ Other

Current gestational age: __________ weeks

Current hospital stay: __________ days

Diagnosis:

☐ Type 2 diabetes mellitus
☐ Gestational diabetes mellitus
☐ Other

INSTRUCTIONS

After reading the cordel, rate each statement and select only one response. Use the following scale:

1 = totally agree
2 = partially agree
3 = neutral
4 = partially disagree
5 = totally disagree

If you are unable to rate an item, leave it blank and explain in the comments section.

EVALUATION OF THE EDUCATIONAL MATERIAL

A. OBJECTIVES

1. The cordel encourages engagement in diabetes mellitus (DM) care.

☐ 1 ☐ 2 ☐ 3 ☐ 4 ☐ 5

2. It uses easy-to-understand language.

☐ 1 ☐ 2 ☐ 3 ☐ 4 ☐ 5

3. It contains information and explanations to guide DM care.

☐ 1 ☐ 2 ☐ 3 ☐ 4 ☐ 5

4. It encourages behavioral changes in daily care related to the disease and pregnancy.

☐ 1 ☐ 2 ☐ 3 ☐ 4 ☐ 5

B. STRUCTURE AND PRESENTATION

5. The cordel-style writing is easy to understand.

☐ 1 ☐ 2 ☐ 3 ☐ 4 ☐ 5

6. The text is clear.

☐ 1 ☐ 2 ☐ 3 ☐ 4 ☐ 5

7. The text is interesting.

☐ 1 ☐ 2 ☐ 3 ☐ 4 ☐ 5

8. The suggested content is appropriate.

☐ 1 ☐ 2 ☐ 3 ☐ 4 ☐ 5

9. The font size is adequate.

☐ 1 ☐ 2 ☐ 3 ☐ 4 ☐ 5

10. The illustrations help to complement the text.

☐ 1 ☐ 2 ☐ 3 ☐ 4 ☐ 5

C. RELEVANCE

11. The material addresses the necessary topics for understanding DM care during pregnancy.

☐ 1 ☐ 2 ☐ 3 ☐ 4 ☐ 5

12. The material is suitable to assist with DM care at home.

☐ 1 ☐ 2 ☐ 3 ☐ 4 ☐ 5

13. It allows reflection on mistakes made, leading to behavioral change.

☐ 1 ☐ 2 ☐ 3 ☐ 4 ☐ 5

14. The use of this technology is relevant for pregnant women with DM.

☐ 1 ☐ 2 ☐ 3 ☐ 4 ☐ 5

COMMENTS AND SUGGESTIONS

What did you like most about the cordel?

What should be modified to make the material clearer, more useful, or more appropriate?

**EVALUATION OF THE EDUCATIONAL CORDEL ON DIABETES DURING PREGNANCY — EXPERTS**

Participant code: ______________________________

Date: **/**/________

Institution/site: ______________________________

Interviewer/evaluator: ______________________________

PARTICIPANT CHARACTERISTICS

Institution of origin:

☐ HGCC ☐ MEAC ☐ Other: ________________________________________

Age: __________ years

Sex:

☐ Female ☐ Male ☐ Other/Prefer not to disclose

Profession:

☐ Obstetric nurse ☐ Obstetrician ☐ Other: _______________________

Year of graduation: __________

Time since graduation: __________ years

Experience in women’s health care:

☐ Up to 2 years
☐ >2–5 years
☐ >5–10 years
☐ >10 years

Highest academic degree:

☐ Specialization
☐ Master’s
☐ Doctorate
☐ Other: ______________________

Main field of practice:

INSTRUCTIONS

After reading the cordel, rate each statement and select only one response. Use the following scale:

1 = totally agree
2 = partially agree
3 = neutral
4 = partially disagree
5 = totally disagree

If you are unable to rate an item, leave it blank and explain in the comments section.

EVALUATION OF THE EDUCATIONAL MATERIAL

A. OBJECTIVES

1. Alignment with target population needs.

☐ 1 ☐ 2 ☐ 3 ☐ 4 ☐ 5

2. Appropriate language for the target population.

☐ 1 ☐ 2 ☐ 3 ☐ 4 ☐ 5

3. Provides information to support self-care practices.

☐ 1 ☐ 2 ☐ 3 ☐ 4 ☐ 5

4. Encourages behavioral changes for self-care.

☐ 1 ☐ 2 ☐ 3 ☐ 4 ☐ 5

5. Visual presentation captures audience interest.

☐ 1 ☐ 2 ☐ 3 ☐ 4 ☐ 5

6. Potential for scientific dissemination.

☐ 1 ☐ 2 ☐ 3 ☐ 4 ☐ 5

7. Alignment with institutional objectives.

☐ 1 ☐ 2 ☐ 3 ☐ 4 ☐ 5

8. Consistency with Ministry of Health recommendations.

☐ 1 ☐ 2 ☐ 3 ☐ 4 ☐ 5

B. STRUCTURE AND PRESENTATION

9. Technology appropriate for pregnant women.

☐ 1 ☐ 2 ☐ 3 ☐ 4 ☐ 5

10. Messages are clear and direct.

☐ 1 ☐ 2 ☐ 3 ☐ 4 ☐ 5

11. Information is scientifically accurate.

☐ 1 ☐ 2 ☐ 3 ☐ 4 ☐ 5

12. Cover is attractive and representative.

☐ 1 ☐ 2 ☐ 3 ☐ 4 ☐ 5

13. Title and text size are appropriate.

☐ 1 ☐ 2 ☐ 3 ☐ 4 ☐ 5

14. Cordel format facilitates understanding.

☐ 1 ☐ 2 ☐ 3 ☐ 4 ☐ 5

15. Logical organization of content.

☐ 1 ☐ 2 ☐ 3 ☐ 4 ☐ 5

16. Font size is appropriate.

☐ 1 ☐ 2 ☐ 3 ☐ 4 ☐ 5

17. Illustrations support the content.

☐ 1 ☐ 2 ☐ 3 ☐ 4 ☐ 5

18. Amount of information is appropriate.

☐ 1 ☐ 2 ☐ 3 ☐ 4 ☐ 5

C. RELEVANCE

19. Covers essential content on gestational diabetes.

☐ 1 ☐ 2 ☐ 3 ☐ 4 ☐ 5

20. Supports nurses in home-based guidance.

☐ 1 ☐ 2 ☐ 3 ☐ 4 ☐ 5

21. Enables post-discharge follow-up.

☐ 1 ☐ 2 ☐ 3 ☐ 4 ☐ 5

22. Aligned with Ministry of Health care guidelines.

☐ 1 ☐ 2 ☐ 3 ☐ 4 ☐ 5

23. Promotes behavioral change.

☐ 1 ☐ 2 ☐ 3 ☐ 4 ☐ 5

24. Suitable as a discharge guidance tool.

☐ 1 ☐ 2 ☐ 3 ☐ 4 ☐ 5

25. Technology is relevant for pregnant women with diabetes.

☐ 1 ☐ 2 ☐ 3 ☐ 4 ☐ 5

COMMENTS AND SUGGESTIONS

Record any technical, scientific, linguistic, or graphic corrections and, whenever possible, provide the suggested wording.

General comments on the applicability and relevance of the material:
